# Site-Specific Immobilization Boosts the Performance of a Galectin-1 Biosensor

**DOI:** 10.1021/acs.bioconjchem.4c00467

**Published:** 2024-12-03

**Authors:** Dajana Kolanovic, Rajeev Pasupuleti, Jakob Wallner, Georg Mlynek, Birgit Wiltschi

**Affiliations:** † acib − Austrian Centre of Industrial Biotechnology, Graz 8010, Austria; ‡ Institute of Molecular Biotechnology, Graz University of Technology, Graz 8010, Austria; § BOKU Core Facility Biomolecular & Cellular Analysis, BOKU University, Vienna 1190, Austria; ∥ Institute of Bioprocess Science and Engineering, Department of Biotechnology, BOKU University, Vienna 1190, Austria

## Abstract

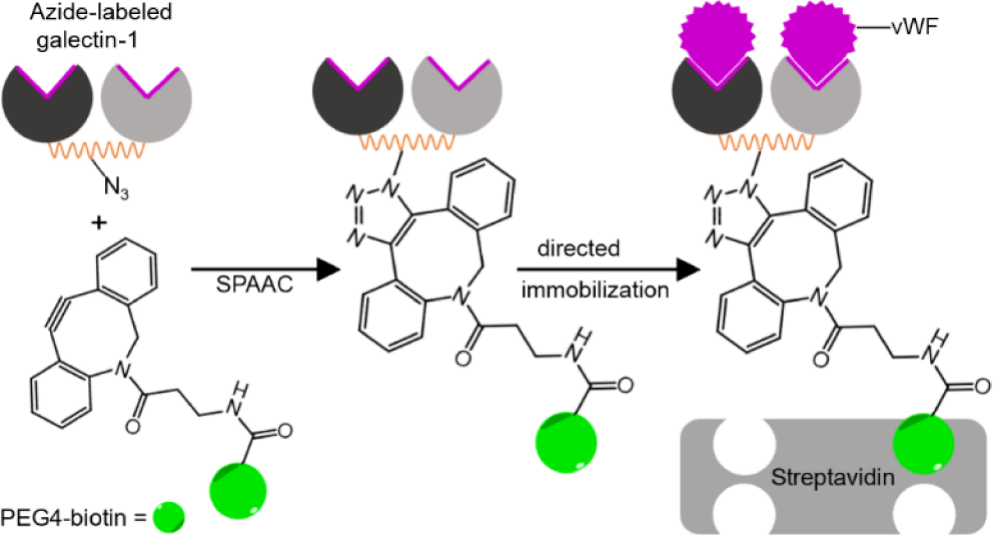

The analysis of protein-bound glycans has gained significant attention due to their pivotal roles in physiological and pathological processes like cell–cell recognition, immune response, and disease progression. Routine methods for glycan analysis are challenged by the very similar physicochemical properties of their carbohydrate components. As an alternative, lectins, which are proteins that specifically bind to glycans, have been integrated into biosensors for glycan detection. However, the effectiveness of protein-based biosensors depends heavily on the immobilization of proteins on the sensor surface. To enhance the sensitivity and/or selectivity of lectin biosensors, it is crucial to immobilize the lectin in an optimal orientation for ligand binding without compromising its function. Random immobilization methods often result in arbitrary orientation and reduced sensitivity. To address this, we explored a directed immobilization strategy relying on a reactive noncanonical amino acid (ncAA) and bioorthogonal chemistry. In this study, we site-specifically incorporated the reactive noncanonical lysine derivative, N^ε^-((2-azidoethoxy)carbonyl)-l-lysine, into a cysteine-less single-chain variant of human galectin-1 (scCSGal-1). The reactive bioorthogonal azide group allowed the directed immobilization of the lectin on a biosensor surface using strain-promoted azide–alkyne cycloaddition. Biolayer interferometry data demonstrated that the controlled, directed attachment of scCSGal-1 to the biosensor surface enhanced the binding sensitivity to glycosylated von Willebrand factor by about 12-fold compared to random immobilization. These findings emphasize the importance of controlled protein orientation in biosensor design. They also highlight the power of single site-specific genetic encoding of reactive ncAAs and bioorthogonal chemistry to improve the performance of lectin-based diagnostic tools.

## 1 Introduction

Saccharides that are covalently attached to proteins or lipids, also known as glycans, have attracted considerable attention in the scientific community during the past decade. Glycans have a role in various physiological and pathological processes, such as intermolecular and cell–cell recognition events. They influence the cell cycle, cell differentiation, apoptosis, host–pathogen interactions, and inflammation.^1,2^ Given that approximately 70–80% of all human proteins are glycosylated and that aberrant glycosylation can indicate the presence of disease, it is not surprising that monitoring changes in protein glycan structures has become a valuable diagnostic tool.^3,4^ The glycans comprise a group of biomolecules of considerable structural variability^5^ although their building blocks, the carbohydrates, possess very similar physicochemical properties. The conformity of the carbohydrates challenges the elucidation of the exact glycan structures using instrumental techniques such as nuclear magnetic resonance (NMR), mass spectrometry, chromatography, or electrophoresis.^6^ Consequently, alternative methods for glycan analysis have emerged, which exploit natural glycan-binding proteins known as lectins.

Lectins are a large family of proteins of nonimmune origin that are ubiquitous in nature. They recognize and bind both free glycans and glycans attached to glycoconjugates, such as glycoproteins and glycolipids. These glycans may be located within cells, attached to cell membranes, or secreted into biological fluids.^7^ Lectins possess complex specificities, enabling them to specifically recognize not only different monosaccharides within the glycan chain but also different linkages between saccharide monomers and glycan branching.^8^ Lectins can be used for glycan analysis directly on glycoproteins and glycolipids or the surface of intact cells and viruses. Since the glycans do not need to be detached from the protein or lipid backbone as is required with instrumental techniques, lectins have become increasingly integrated into various biosensor designs.^9,10^ However, there are challenges in the development and application of lectin biosensors for diagnostic purposes. To make lectin biosensors a viable diagnostic tool for detecting low levels of disease biomarkers, which is essential for early stage diagnostics, they must offer high specificity as well as sensitivity of detection. For selective and sensitive detection in a reproducible manner, it is crucial that the protein molecules are immobilized on the biosensor surface in a highly controllable manner, ensuring optimal orientation for ligand binding without compromising their activity.

Various protein immobilization strategies have been developed, each with its own advantages and limitations. Classic, nonspecific immobilization methods most often rely on functional groups present in the side chains of amino acids. Conjugation at, for instance, the epsilon-amino group of lysine residues or the thiol group of cysteine, two amino acids occurring frequently in proteins, results in random protein orientation, which can compromise functionality and reduce biosensor sensitivity.^11,12^ To address this challenge, site-specific immobilization techniques have emerged. These methods enable controlled coupling of protein molecules to the sensor surface, ensuring that their active sites are optimally positioned for interaction with the ligand. This precise orientation significantly enhances the sensitivity and selectivity of biosensors, making them more reliable for diagnostic applications.^13,14^

To achieve site-specific immobilization of a protein on the sensor surface, a unique functional group has to be introduced into the protein at a specific site. Ideally, this functionality should be bioorthogonal, which means it does not naturally occur in the amino acid building blocks of proteins or in other bio(macro)molecules and does not cross-react with endogenous functional groups. The presence of such a unique, bioorthogonal functional group at one defined site in the protein enables its selective and site-specific coupling to a complementary reactive group on the sensor surface. Bioorthogonal chemistries such as copper(I)-catalyzed or strain-promoted azide–alkyne cycloaddition (CuAAC and SPAAC, respectively), so-called “click” chemistries, have demonstrated their utility and efficacy for protein modifications.^15−17^ Neither azide nor alkyne moieties occur naturally in recombinant proteins; consequently, techniques to introduce them at any position of choice have become of great interest.

There are several chemical and biological techniques to introduce bioorthogonal reactive groups into proteins. Chemical approaches use bifunctional linkers that carry both a bioorthogonal group and a chemical group for conjugation with reactive side chains of canonical amino acids, usually lysines or cysteines. This approach offers limited options for site-specific installation of a bioorthogonal group in the protein, as it relies on the presence of a single lysine or cysteine at the desired position. Additionally, the modification of lysines or cysteines that are essential for biological activity can impair the function of the protein (reviewed by de Graaf et al.^18,19^). In contrast, the genetic encoding of the bioorthogonal groups offers unprecedented control over their incorporation position in the protein. Noncanonical amino acids (ncAAs) carrying bioorthogonal reactive side chain moieties such as azide or alkyne groups are not encoded by the standard genetic code. Nevertheless, they can be incorporated by ribosomal protein translation under tightly controlled conditions, e.g., in response to an in-frame amber codon. The in-frame amber codon is decoded by a suppressor tRNA_CUA_, which is charged with the reactive ncAA by an aminoacyl-tRNA synthetase (aaRS). While wild-type aaRSs with the ability to recognize and charge the ncAA onto the tRNA_CUA_ exist,^20,21^ mutant aaRSs are often employed due to their enhanced efficiency and specificity in charging ncAAs.^22,23^ Moreover, the aaRS/tRNA_CUA_ pair must be orthogonal in the host organism. This means that the aaRS does not charge any of the host tRNAs with the ncAA, nor is the tRNA_CUA_ charged with a canonical amino acid by any of the host aaRSs.^24^ This method, known as’stop codon suppression’ (SCS), allows the genetic encoding of a bioorthogonal group at a strategically chosen position in the gene of interest. As such, SCS facilitates the generation of site-specifically functionalized proteins for the controlled and oriented immobilization on sensor surfaces. The homogeneously accessible orientation of the ligand-binding sites of all sensor proteins on the surface can improve the biosensor’s overall performance.^11^ Genetic code expansion technologies enabling the incorporation of ncAAs are well-established and have been utilized for numerous applications, including protein immobilization. In fact, ncAAs have been employed for protein immobilization for nearly two decades.^25−28^

In this study, we explored the effectiveness of directed immobilization by the genetic encoding of a bioorthogonally reactive ncAA combined with click chemistry to enhance the sensitivity of a lectin biosensor. We compared this approach with the classic immobilization at lysine residues to assess whether the controlled protein orientation would improve the biosensor’s performance. As a model lectin in this study, we chose a cysteine-less mutant of human galectin-1 (CSGal-1). Galectin-1, a conserved member of the lectin family, binds β-galactoside moieties on glycoproteins and glycolipids. It plays a significant role in modulating cell adhesion, immune response, and tumor progression. Galectin-1 contains one carbohydrate recognition domain (CRD) per subunit and typically homodimerizes noncovalently in solution.^29−31^ A reactive ncAA that is incorporated at a selected position in the monomer would be present in both subunits of the homodimer. The presence of two reactive handles can complicate the immobilization process, potentially leading to aberrant orientation and suboptimal performance of the biosensor.^32^ To address the issues associated with the noncovalent dimeric form of galectin-1, we generated a single-chain (sc) version of CSGal-1 by genetically fusing its subunits with a *de novo* designed peptide linker (manuscript in preparation). This protein architecture ensures that the ncAA incorporation occurs at a single, genetically determined position within a monomeric protein, which retains its two CRDs.

Von Willebrand factor (vWF), a multimeric glycoprotein essential for blood coagulation and platelet aggregation, has been identified as a binding partner for galectin-1.^33^ vWF protein binds to receptors on the surface of platelets and within connective tissue and facilitates platelet adhesion at sites of vascular injury. Under these conditions, vWF uncoils and decelerates passing platelets. Deficiency or dysfunction of vWF, which leads to von Willebrand disease (VWD), is characterized by bleeding symptoms such as frequent nosebleeds, easy bruising, and excessive bleeding from minor injuries.^34^ Additionally, vWF is implicated in various other conditions, including thrombosis, stroke, and Heyde’s syndrome.^35,36^ An enhanced sensitivity to detect the galectin-1-vWF interaction holds great promise for improved diagnostic assays for various diseases in which vWF plays a crucial role.

Toward this end, we employed the SCS method with the orthogonal pyrrolysyl-tRNA synthetase (PylRS)/tRNA_CUA_^Pyl^ pair from *Methanosarcina mazei* (*Mm*) and site-specifically incorporated the unique reactive ncAA, N^ε^-((2-azidoethoxy)carbonyl)-l-lysine (AzK; Figure S1) at selected positions into the scCSGal-1 protein. scCSGal-1 AzK variants were site-specifically conjugated with an alkyne-biotin linker using SPAAC. For comparison, we randomly biotinylated scCSGal-1 wild-type using standard *N*-hydroxysuccinimide (NHS) ester coupling. Site-specifically and randomly biotinylated scCSGal-1 proteins were immobilized on a surface covered with streptavidin, to which biotin binds near covalently.^37^ To evaluate and compare the effectiveness of this site-specific immobilization strategy against the standard randomly oriented coupling method, we employed Bio-Layer Interferometry (BLI), an optical analytical technique. BLI enables real-time, label-free analysis of biomolecular interactions, making it an ideal biosensor platform to assess the binding sensitivity between immobilized scCSGal-1 and its binding partner, glycoprotein vWF.

We found that the directed immobilization of scCSGal-1 by bioorthogonal coupling *via* a reactive ncAA significantly enhanced the biosensor’s sensitivity toward vWF in comparison to classic random immobilization. This finding not only underscores the importance of protein orientation in lectin biosensor design. It also highlights the potential of controlled genetic encoding of ncAAs carrying reactive groups for bioorthogonal chemistry to devise high-performance protein biosensors.

## 2 Results and Discussion

### 2.1 Construction of Single-Chain CSGal-1 for Immobilization

**Figure 1:**
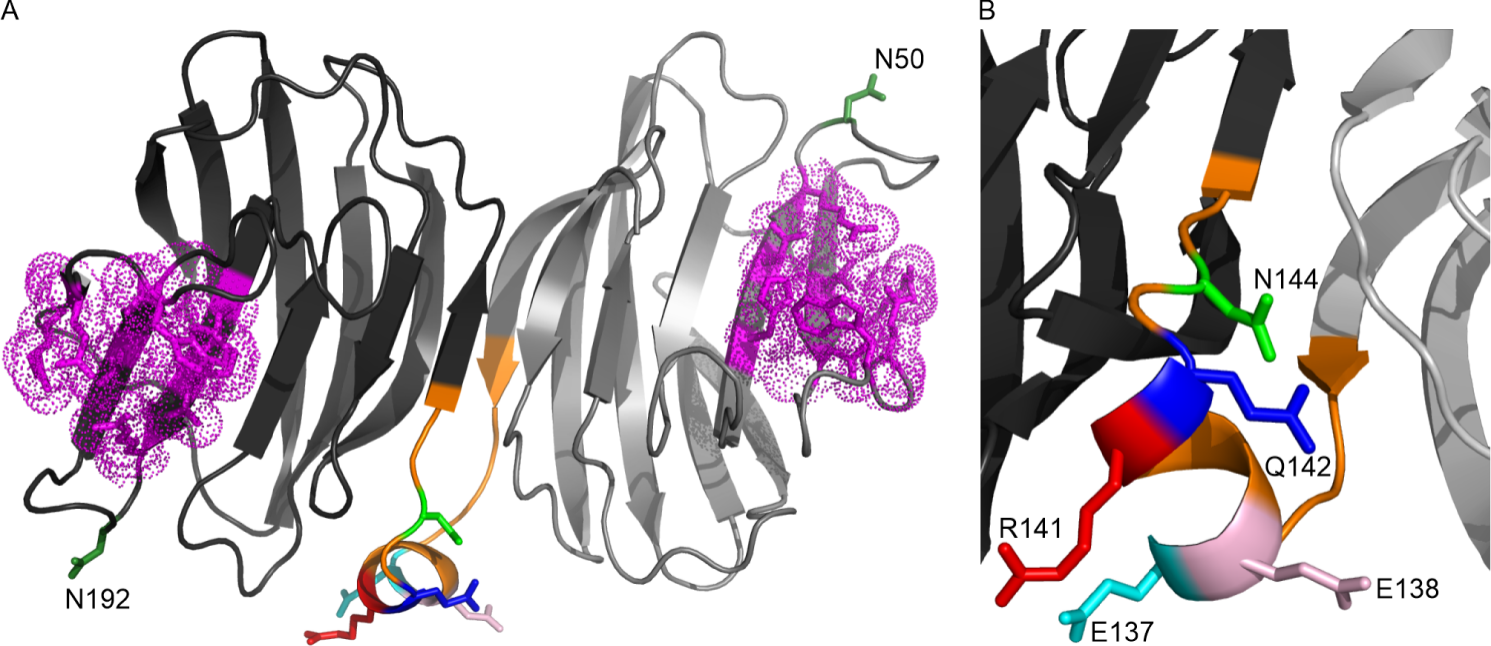
Predicted structure of single-chain (sc) human CSGal-1 showing selected positions for AzK incorporation. (A) The structure displays the two monomers in dark and light gray cartoons, connected by an orange linker. The glycan binding site of each monomer is depicted as sticks and dots in magenta. Selected positions for AzK incorporation are illustrated as sticks: E137 in cyan, E138 in light pink, R141 in red, Q142 in blue, and N144 in green, while alternative positions N50 and N192 are shown in dark green. (B) A close-up view of the selected positions introduced in the linker region. For purification purposes, a 6H-tag (including GS linker) was added to the C-terminus of scCSGal-1 (not shown here, see Table S1).

To select appropriate positions for the immobilization of the single-chain (sc) protein of the cysteine-less (CS) mutant of human galectin-1 (Gal-1) (scCSGal-1), we analyzed its predicted 3D structure (Figure 1A). We sought to identify surface-exposed residues that are not located in or near the binding sites to avoid binding disturbances. Instead, we targeted residues located opposite of the scCSGal-1 binding sites to enable immobilization in a way that would expose both binding sites to the ligand. By following these criteria, we identified five surface-exposed positions in scCSGal-1 as candidates for incorporating AzK and subsequent immobilization. The five positions E137, E138, R141, Q142, and N144 were selected in the linker sequence that connects the two CSGal-1 monomers (Figure 1B). This linker region, which is distant and opposite to both glycan binding sites, served our immobilization purpose perfectly. Multiple sites were chosen because the incorporation of an ncAA can affect the expression and/or functionality of a protein in a sequence-dependent manner. Screening of several incorporation sites for their permissiveness toward ncAA incorporation usually increases success.^38^ Further, two other surface-exposed positions, N50 and N192, were selected as alternative positions outside of the linker sequence (Figure 1A). These residues are not involved in the glycan binding, so their substitution should not affect the binding activity. However, N50 and N192 are located on the same face of the protein as the binding sites, which may allow exposure of only one binding site to the ligand and not both.

For protein purification purposes, we added a hexahistidine (6H)-tag (including GS linker) to the C-terminus of scCSGal-1 as described in the materials and methods section. The C-terminal 6H-tag enables purification by immobilized metal affinity chromatography on a Zn^2+^-sepharose matrix (Zn-IMAC). As it is expressed only when readthrough at the in-frame amber codon occurs, it ensures that only full-length scCSGal-1–6H AzK variants are recovered as further detailed below. However, the addition of a C-terminal 6H-tag can affect protein functionality.^39^ Therefore, we produced scCSGal-1–6H and assessed its functionality using lactose-affinity chromatography (Figure S2A). Bioactive human galectin-1 binds β-galactosides, such as lactose,^40^ so only functional protein binds to the lactose column and can be eluted with free lactose. Sodium dodecyl sulfate polyacrylamide gel electrophoresis (SDS-PAGE) analysis demonstrated that scCSGal-1–6H efficiently bound to and was subsequently eluted from the lactose-affinity matrix (Figure S2A, black arrow). This finding revealed that scCSGal-1–6H retained its functional conformation despite the addition of the C-terminal 6H-tag. The purified protein migrated at an apparent molecular weight >35 kDa on an SDS-PA gel, which was higher than its calculated molecular weight (MW_calc_) of ∼31 kDa. We observed this aberrant protein migration on SDS-PA gels has in a previous study (manuscript in preparation) and attributed it to the high content of acidic amino acids in the scCSGal-1 protein. Surprisingly, in the purified samples, we observed an additional band at >15 kDa corresponding to a truncated but functional scCSGal-1–6H (Figure S2A, white arrow). It indicates that the truncated protein comprises approximately half of scCSGal-1–6H and retains at least one glycan binding site. To explore the truncated protein in more detail, we conducted a thorough *in silico* analysis of the *scCSGal-1–6H* gene sequence (Table S1). We identified an internal Shine-Dalgarno (SD) sequence “GGAGG” in the middle of the *scCSGal-1–6H* gene (Table S1), followed by an ATG sequence that occurred 11 bases downstream of this internal SD sequence. It is likely that host ribosomes bound to the internal SD sequence and initiated translation, which resulted in the truncated protein containing the second half of the scCSGal-1–6H with one binding site and a C-terminal 6H-tag. To verify this theory, we repurified the lactose-purified protein sample using Zn-IMAC (Figure S2B). Indeed, SDS-PAGE analysis revealed that not only the full-length protein (Figure S2B, black arrow) but also a truncated protein could be copurified by Zn-IMAC, which confirmed the presence of the C-terminal 6H-tag (Figure S2B, white arrow). To eliminate any potential interference by the truncated scCSGal-1 in the downstream experiments, we mutated the ATG sequence to ATC, changing the methionine (M) codon to a leucine (L) codon. Leucine is structurally similar to methionine in terms of size and hydrophobicity, making it a suitable replacement that most likely preserves the overall protein structure and function.^41^ We reasoned that this mutation would prevent the “accidental” translation initiation, ensuring that only full-length proteins would be expressed and purified. The mutated protein, scCSGal-1–6H M120L, was produced and purified using Zn-IMAC followed by lactose affinity chromatography (Figure S3). As expected, no truncated proteins were visible on the SDS-PA gel, and only full-length proteins were purified. SDS-PAGE analysis also confirmed that the M120L mutation preserved the functionality of the protein, which was evidenced by its binding to the lactose affinity column. Consequently, this full-length and functional protein, scCSGal-1–6H M120L, was used to introduce azido groups at the selected positions.

### 2.2 scCSGal-1–6H M120L AzK Variants Were Successfully Produced

**Figure 2:**
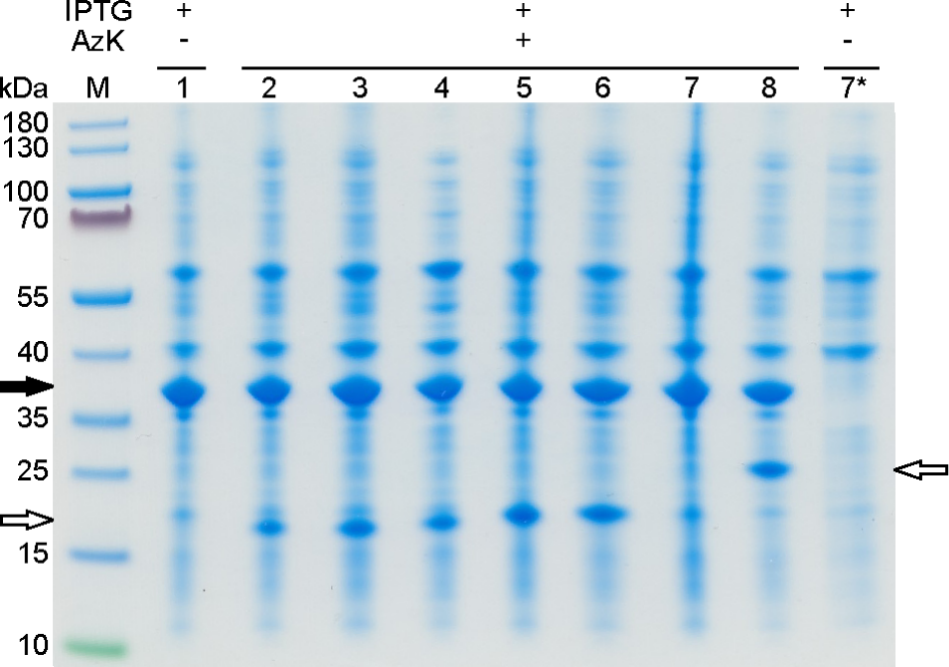
Expression study of scCSGal-1–6H M120L amber mutants in the presence of AzK in *E. coli* BL21(DE3). Seven potential positions (see Figure 1) were selected to incorporate AzK into scCSGal-1–6H M120L. Whole cell samples were collected 18 h after addition of AzK during IPTG induction. All seven scCSGal-1–6H M120L azido protein variants were clearly produced in the presence of AzK (lanes 2–8). There was no significant variation in expression levels between the AzK variants or when compared to the wild-type protein (lane 1). On the other hand, when AzK was not supplemented to the culture medium during IPTG induction, a full-length scCSGal-1–6H M120L N50AzK variant was not produced (lane 7*). The truncated protein bands were observed for all variants (lanes 2–6, lane 8), except for N50am (lane 7). The truncated N50am mutant was most probably not detectable because the termination of translation occurred only 50 codons from the N-terminus, which resulted in a very small, 5.4 kDa truncation fragment. Lane M, molecular size marker; lane 1, scCSGal-1–6H M120L wt; lanes 2–8, scCSGal-1–6H M120L variants: E137AzK (lane 2), E138AzK (lane 3), R141AzK (lane 4), Q142AzK (lane 5), N144AzK (lane 6), N50AzK (lane 7), and N192AzK (lane 8).The numbers on the left margin of the gel represent the size of the molecular weight marker bands in kDa. The MW_calc_ of scCSGal-1–6H M120L wt and its AzK variants (black arrow) is ∼31 kDa, which migrate at >35 kDa on the Coomassie stained 4–12% Bis-Tris SDS-PA gel. The white arrows indicate truncated proteins of AzK variants with a MW_calc_ of 14.7 kDa, 14.8 kDa, 15.2 kDa, 15.3 kDa, 15.5 kDa, and 20.7 kDa in lanes 2–6 and 8, respectively.

To functionalize scCSGal-1–6H M120L with an azide-reactive handle for directed immobilization *via* incorporation of the ncAA AzK, we used the orthogonal (o) *Mm*PylRS/*Mm*tRNA_CUA_^Pyl^ pair. The *Mm*PylRS charges the *Mm*tRNA_CUA_^Pyl^ with a palette of (pyro)lysine derivatives, such as AzK. The charged tRNA_CUA_^Pyl^ decodes the in-frame amber stop codon UAG on the mRNA and incorporates AzK or any other (pyro)lysine derivative into the growing nascent polypeptide chain during translation.^23,42^ To introduce an amber codon (TAG) at the selected positions for AzK incorporation in scCSGal-1–6H M120L, we performed overlap extension PCR, as described in the materials and methods section. The resulting inserts, *scCSGal-1–6H M120L* with an in-frame amber (am) stop codon at position E137, E138, R141, Q142, N144, N50 or N192 were introduced into the pT7 × 31 expression vector. In this vector, the expression of the target protein, the *Mm*PylRS, and the suppressor *Mm*tRNA_CUA_^Pyl^ are controlled by the T7 promoter,^43^ facilitating improved ncAA incorporation. The *E. coli* BL21(DE3) expression strain was transformed with the constructed expression vectors and cultivated overnight at 20 °C with vigorous shaking. To analyze whether the location of the ncAA incorporation site affected the expression of the scCSGal-1–6H M120L amber mutants, we collected cell samples 18 h after addition of AzK during IPTG induction (Figure 2). The wild-type (wt) gene, *scCSGal-1–6H M120L wt*, was expressed as a benchmark control (Figure 2, lane 1) and the collected samples were analyzed by SDS-PAGE. On the SDS-PA gel, we observed that SCS with AzK produced all positional variants of scCSGal-1–6H M120L (Figure 2, black arrow, lanes 2–8) without significant variation in their expression levels across different mutation sites or compared to the wild-type protein (Figure 2, black arrow, lane 1). As a negative control, we used the scCSGal-1–6H M120L N50am amber mutant and induced recombinant gene expression with IPTG but without adding AzK to the culture medium. This amber mutant was not expressed, which confirmed the absence of background incorporation of canonical amino acids with the *Mm* o-pair (Figure 2, lane 7*). However, we observed truncated versions of all amber mutants (white arrows, Figure 2, lanes 2–6 and lane 8), as a result of unsuppressed amber stop codon and premature termination of translation. This finding indicated that incorporation with the *Mm* o-pair was specific yet not quantitative. For N50am, the truncated protein was not detected (Figure 2, lane 7) because the amber codon is positioned only 50 codons from the N-terminus. The resulting truncated protein had a MW_calc_ of only 5.4 kDa, which was so small that it most probably migrated in the dye front on the SDS-PA gel as indicated by the protein marker bands used in this study. To eliminate interference from truncated proteins and purify only full-length variants, we employed Zn-IMAC purification. This straightforward approach was expected to isolate exclusively full-length proteins. Truncated proteins resulting from unsuppressed amber codons lack the C-terminal 6H-tag necessary for Zn-IMAC purification and thus cannot be isolated by this method. Our results confirmed this expectation, as no truncated protein bands were observed in Figure S4 (white arrows, lanes 2–8). Furthermore, all eight proteins were successfully purified by affinity chromatography on a lactose–agarose resin, which confirmed their functional ligand binding. The titers of the purified soluble AzK variants ranged from 9.8 to 10.7 mg per liter of culture medium, which is comparable to the wild-type protein titer of 11.1 mg per liter of culture.

### 2.3 AzK was Successfully Incorporated in scCSGal-1–6H M120L

**Figure 3:**
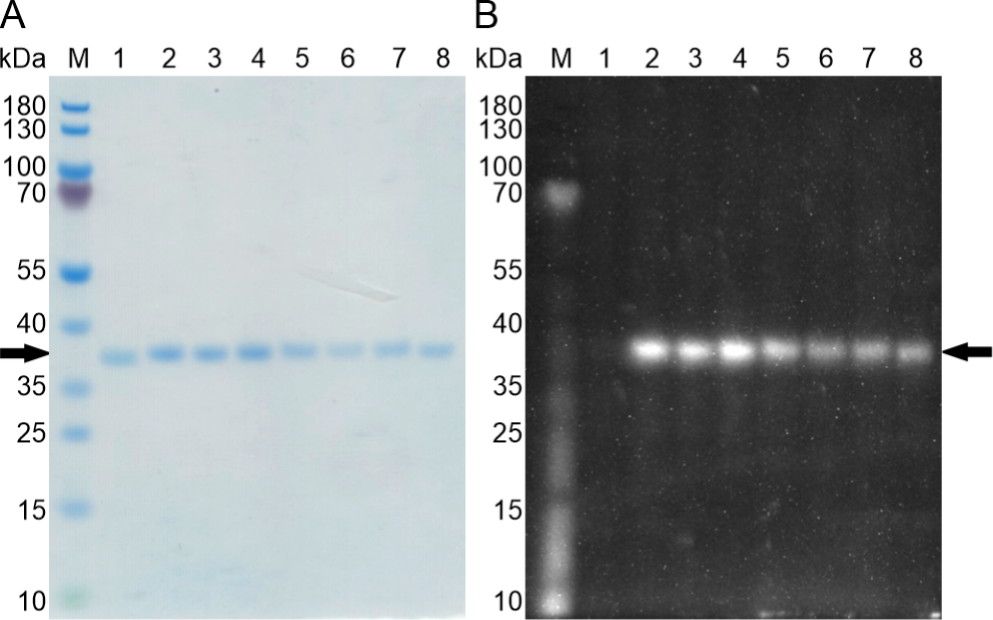
Confirmation of AzK incorporation in scCSGal-1–6H M120L azido-variants by fluorophore labeling *via* SPAAC. Following Zn-IMAC purification (Figure S4), lactose-purified full-length proteins, scCSGal-1–6H M120L wt and its seven AzK variants (lanes 1–8), were incubated with DBCO-Cy3 fluorophore and then analyzed by SDS-PAGE. (A) The proteins were stained with Coomassie stain. (B) The SDS-PA gel, exposed to UV light before Coomassie staining, shows fluorescent bands resulting from the incubation of AzK variants with DBCO-Cy3 (lanes 2–8). This confirms the presence of the azide group in scCSGal-1–6H M120L AzK variants, which is required for successful conjugation with the alkyne-containing fluorophore. The negative control, scCSGal-1–6H M120L wt, which lacks an azide reactive group for bioorthogonal conjugation, did not display a fluorescent band with the DBCO-Cy3 fluorophore confirming the specificity of the conjugation reaction. Lane M, molecular size marker; lane 1, scCSGal-1–6H M120L wt; lanes 2–8, scCSGal-1–6H M120L variants: E137AzK (lane 2), E138AzK (lane 3), R141AzK (lane 4), Q142AzK (lane 5), N144AzK (lane 6), N50AzK (lane 7), and N192AzK (lane 8). The numbers on the left margins of the gels indicate the size of the molecular weight marker bands in kDa. The black arrows indicate scCSGal-1–6H M120L wt and its AzK variants (MW_calc_ ∼ 31 kDa).

To verify the incorporation of AzK at selected positions in the scCSGal-1–6H M120L azido-variants, we conjugated the lactose-purified proteins with the dibenzocyclooctyne-sulfo-Cyanine-3 (DBCO-Cy3) fluorophore *via* strain-promoted azide–alkyne cycloaddition (SPAAC). The SPAAC reaction, also known as a “click reaction,” is a cycloaddition of a cyclic alkyne (e.g., DBCO) and an organic azide (e.g., AzK), resulting in a stable triazole product. The reaction is spontaneous, highly efficient, regioselective, and can be performed under mild conditions at room temperature.^44^ The wild-type protein scCSGal-1–6H M120L, which did not contain an azide reactive group for bioorthogonal conjugation, was used as a negative control to assess nonspecific conjugation. The reaction mixtures were then separated on the SDS-PA gel and analyzed by UV irradiation before Coomassie staining (Figure 3). As expected, the scCSGal-1–6H M120L AzK proteins displayed a fluorescent band on the SDS-PA gel, indicating the presence of azide groups and their successful conjugation with the alkyne-containing fluorophore (Figure 3B, lanes 2–8). This confirmed both the successful incorporation of AzK into the scCSGal-1–6H M120L azido variants and the accessibility of the reactive azide group for conjugation. Furthermore, the analysis demonstrated that the SPAAC reaction was consistently efficient across all AzK variants, independent of their individual AzK positions. We did not observe noticeable differences in conjugation efficiency among the distinct local environments of the AzK residues. In contrast, the scCSGal-1–6H M120L wild-type protein, which lacks AzK, did not show a fluorescent band with DBCO-Cy3, demonstrating no nonspecific binding of the fluorophore (Figure 3B, lane 1).

Mass spectrometry (MS) analysis of the intact proteins further confirmed the incorporation of AzK in all scCSGal-1–6H M120L azido variants (Figure S5). The mass difference between the calculated and observed masses of the protein samples was ≤0.2 Da (Table S2).

### 2.4 The scCSGal-1–6H M120L AzK Variants Retained Their Glycan Binding Properties

The successful purification of the scCSGal-1–6H M120L AzK variants using lactose-affinity chromatography corroborated their functionality (Figure 3). This finding was in line with our expectations, since none of the seven selected AzK positions were involved in ligand binding. However, even if the ncAA was not directly incorporated into the binding site of the protein, ligand binding affinity or specificity could still have been altered.^45^ Changes in distant regions of the protein caused by the presence of the ncAA could allosterically affect the binding site, altering its conformation and thus its affinity for ligands.^46^ To tackle this possibility, we investigated whether the incorporation of AzK affected the glycan binding affinity and specificity of the scCSGal-1–6H M120L AzK variants.

To estimate and compare the binding affinity of AzK-substituted variants, we performed differential scanning fluorimetry (DSF) experiments. Wild-type scCSGal-1–6H M120L, which did not contain AzK, was included as the positive control. DSF experiments were conducted using IMAC-purified scCSGal-1–6H M120L wt and its AzK variants. We did not perform lactose affinity purification at this point to avoid saturation of the CRD with excess lactose during the elution step. Each protein was tested with increasing concentrations of the ligand lactose, ranging from 0 to 160 mM. It is known that ligand binding increases the melting temperature (T_m_) of a protein, which is then detected as a shift in DSF experiments.^47^ Fitting the T_m_ shift as a function of ligand concentration enables the evaluation of ligand binding affinity (*K*_d_). Quantification of *K*_d_ from the unfolding curves at varying ligand concentrations and temperatures yields an approximated value, the so-called “apparent *K*_d_” (*K*_d, app_), which does not consider the temperature dependency of the binding constant. To determine the *K*_d, app_ of the scCSGal-1–6H M120L wt and AzK variants, we fitted the observed melting temperatures (T_mObs_) against varying lactose concentrations using the “Tm shift fitting” model of the Foldaffinity webserver (https://spc.embl-hamburg.de/app/foldAffinity)^48^ (Figure S6). The results demonstrated that the *K*_d, app_ of scCSGal-1–6H M120L AzK variants were in the range of approximately 1–10 mM (Table S3), which is in the same order of magnitude as the *K*_d, app_ of wt protein (*K*_d, app_ = 3.1 mM, Table S3). This finding revealed that the azido-variants had similar binding affinities as the wt, indicating that AzK incorporation at the selected positions did not interfere with the binding site or the overall protein structure required for binding.

As a negative control, we conducted DSF experiments with increasing concentrations of sucrose, a nonligand for galectin-1.^49^ In the presence of sucrose, we did not observe a T_m_ shift with any of the proteins (data not shown), which confirmed their lactose-binding specificity. Taken together, AzK incorporation within a scCSGal-1–6H M120L resulted in retention of ligand binding functionality, affinity, and specificity, implying that the selected AzK positions did not alter the protein’s carbohydrate-binding properties.

### 2.5 Oriented Immobilization Enhanced the Binding Efficiency of scCSGal-1–6H M120L

After confirming that AzK incorporation did not affect the binding affinities of the scCSGal-1–6H M120L AzK proteins, our next objective was to immobilize these variants on a solid support. We intended to scrutinize whether the directed, selective single-site immobilization at AzK improved ligand binding sensitivity compared to a conventional nonselective multisite immobilization, e.g., at amino groups. Previous studies have demonstrated that oriented surface immobilization mediated by ncAAs could enhance the performance of proteins in biosensing, biocatalysis, and other applications that rely on protein immobilization. For instance, Seo et al. site-specifically incorporated the ncAA ρ-azido-l-phenylalanine into DrrA, a pathogenic protein from *Legionella pneumophila*. By employing click chemistry, they site-specifically biotinylated DrrA through the ncAA and subsequently immobilized it on a streptavidin-coated surface plasmon resonance (SPR) chip. SPR analysis demonstrated that the oriented immobilization increased the binding affinity of DrrA by approximately 10-fold compared to random immobilization.^11^ Similarly, Trilling et al. employed ncAA incorporation with the SPAAC reaction for the oriented immobilization of antibody fragments. They functionalized the variable domain of a llama heavy-chain antibody with a single azidohomoalanine residue and conjugated it to a cyclooctyne-modified SPR chip. The oriented immobilization resulted in a substantial increase in biosensor sensitivity compared to SPR chips coated with randomly immobilized antibodies.^50^ Wu et al. demonstrated that controlling the orientation of immobilized proteins can also be useful in improving the performance of enzymes. By using ncAA incorporation and click chemistry, they achieved directed immobilization of the model enzyme T4 lysozyme. Their study revealed that oriented immobilization enhanced enzyme activity and stability compared to traditional random immobilization methods. Specifically, the oriented immobilized enzyme exhibited 50% and 73% higher activity than the randomly immobilized enzyme after freeze–thaw and chemical denaturation treatments, respectively.^51^ While these studies have demonstrated the benefits of ncAA-based immobilization, to the best of our knowledge, this approach has not yet been studied in the context of lectins. Traditionally, lectins have been randomly immobilized on solid supports.^52^ Specifically, for the construction of biosensors, lectins have been mainly immobilized through self-assembled monolayers (SAMs) and amine coupling.^53−57^ However, to overcome the disadvantages of random immobilization, researchers have turned to genetic fusion of lectins with specific binding tags to achieve oriented immobilization as an important prerequisite for sensitive and selective glycan measurements.

For example, Mahal’s laboratory fused a glutathione S-transferase (GST) tag with seven recombinant bacterial lectins. The N-terminal GST-tagged lectins were immobilized onto glutathione (GSH)-coated slide surfaces through specific GST-GSH binding. The oriented immobilization of GST-tagged lectins led to increased binding affinity compared to random immobilization, as evidenced by fluorescent lectin microarrays.^58^ In another study, rBC2LCN lectin was genetically fused with polystyrene-binding peptides (PS-tags) at the C-terminus for oriented immobilization on polystyrene microplates. The oriented immobilization of PS-tagged rBC2LCN resulted in a 2-fold lower limit of detection than that achieved with randomly immobilized rBC2LCN.^59^ However, genetic fusions typically restrict conjugation to either the N-terminus or C-terminus of proteins, which limits the ability to perform site-selective conjugations. Consequently, this approach prevents the selection of optimal attachment points, which is essential for maximizing the accessibility of the lectins’ carbohydrate-binding sites to glycans and enhancing biosensor performance. Furthermore, fusion tags may adversely affect the folding, activity, and expression of target proteins.^60−62^ Importantly, genetic fusion does not permit the conjugation of proteins with nonprotein biomolecules, such as nucleic acids, lipids, or small molecules. To address the limitations associated with genetic fusions, we aimed to exploit genetic code expansion and click chemistry for the oriented and controlled immobilization of the scCSGal-1–6H M120L lectin.

**Figure 4:**
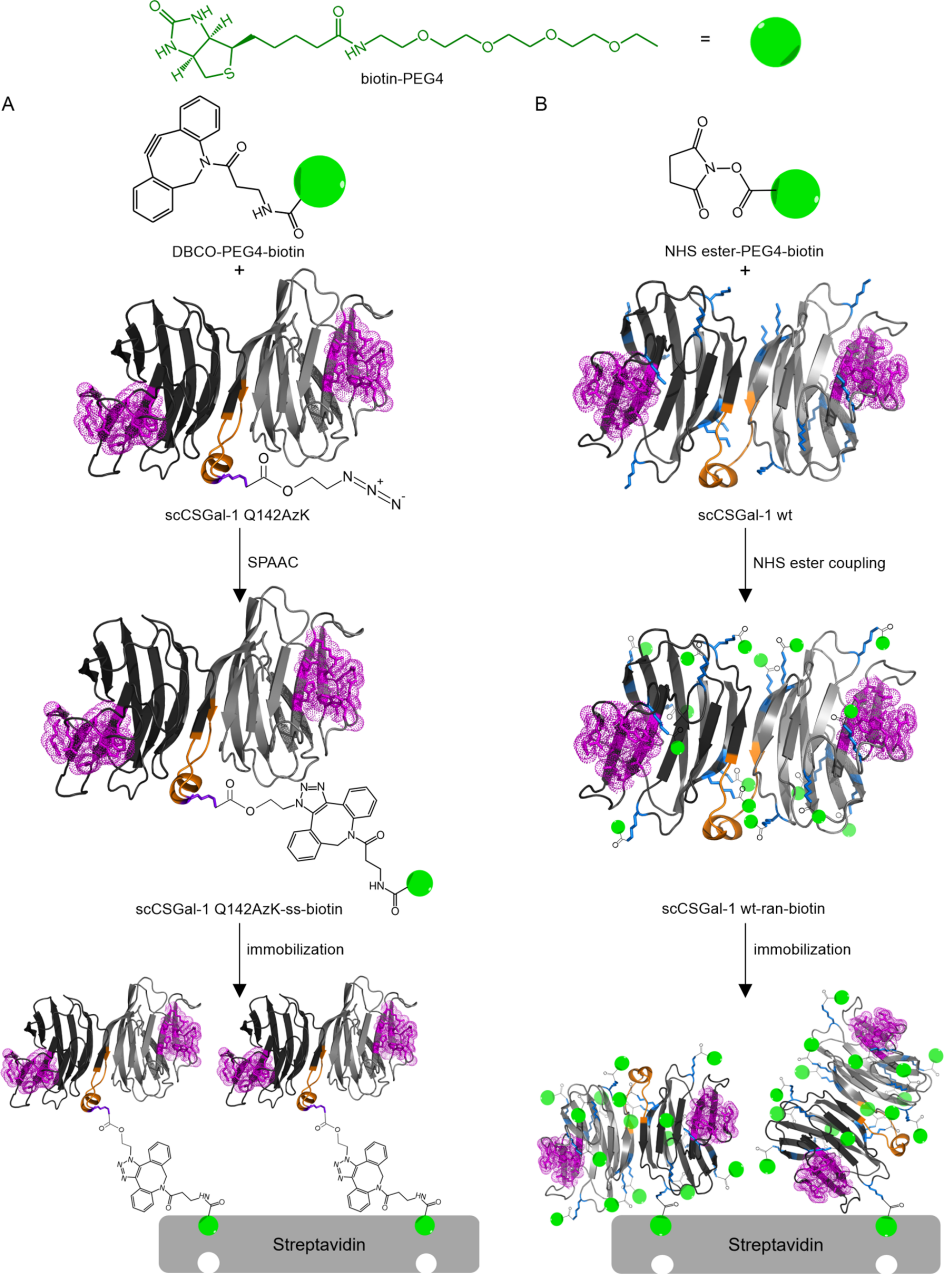
Schematic representation of the immobilization approach. (A) Site-specific (ss) biotinylation of scCSGal-1 Q142AzK *via* SPAAC, resulting in oriented immobilization on the streptavidin surface. (B) Random (ran) biotinylation of scCSGal-1 wt using NHS ester coupling, leading to nonoriented immobilization on the streptavidin surface.

For this purpose, we conjugated biotin to AzK-functionalized scCSGal-1–6H M120L variants by SPAAC between the azide group in the respective AzK variants and the alkyne group in the DBCO-PEG4-biotin linker, as described in the materials and methods section. The unreacted biotin linker was removed from the reaction mixtures using gel filtration. Being an orthogonal, highly efficient, and selective reaction without the necessity of a catalyst, SPAAC has established itself as a broadly applicable chemistry for protein modifications.^63,64^ Since the analysis of the SPAAC reaction with DBCO-Cy3 (Figure 3) indicated that the conjugation efficiency remained consistent irrespective of the AzK position, we did not specifically assess the efficiency of the SPAAC reaction with DBCO-PEG4-biotin for each individual AzK variant. Although DBCO-Cy3 and DBCO-PEG4-biotin are not identical small molecules and their SPAAC efficiencies might differ, we assumed that the efficiencies across individual AzK positions would be comparable. Through this selective conjugation, we site-specifically (ss) biotinylated the scCSGal-1–6H M120L AzK proteins with an alkyne-biotin linker, resulting in scCSGal-1–6H M120L AzK-ss-bio. The scCSGal-1–6H M120L AzK-ss-bio conjugates, each with a single biotin at the intended position, bound to the compatible SA surface in a controlled and oriented manner (Figure 4A). While SPAAC is highly efficient, it is not necessarily 100% efficient under all conditions,^44^ which might have resulted in a heterogeneous mixture of biotinylated and nonbiotinylated scCSGal-1–6H M120L AzK variants. Nevertheless, the protein layer that was formed on the streptavidin (SA) surface was homogeneous because nonbiotinylated scCSGal-1–6H M120L AzK could not bind.

For comparison, we generated randomly (ran) biotinylated scCSGal-1–6H M120L wt, resulting in scCSGal-1–6H M120L wt-ran-bio. This was achieved by coupling biotin to scCSGal-1–6H M120L wt using NHS ester conjugation with an NHS ester-PEG4-biotin linker as described in the materials and methods section. NHS ester reacts specifically and efficiently with primary amine groups that are present on lysine side chains and at the N-terminus of each polypeptide chain.^65^ However, IMAC purification resulted in the copurification of scCSGal-1–6H M120L wt along with other *E. coli* host proteins (Figure S4), which could also potentially react with the NHS ester linker. To avoid interference of these impurities in the further study, we repurified the protein sample using size exclusion chromatography (SEC) (data not shown) and used it for coupling with the NHS ester linker. In scCSGal-1–6H M120L, there are 17 primary amines, including 16 lysine residues and the N-terminus (Table S1), which are potential targets for the biotin coupling reaction. This coupling method was expected to result in heterogeneous scCSGal-1–6H M120L wt-ran-bio proteins with varying numbers of biotins conjugated at random positions, consequently leading to immobilization with random orientations (Figure 4B). We did not assess the number of randomly attached biotins as mass analysis of such complex protein conjugates is notoriously messy. While it is theoretically possible to fine-tune the reaction conditions so that mixtures with reduced biotin functionalities, e.g., 1–2 biotins per protein are obtained, such precision requires extensive optimization. Even if successful, the exact positions of the biotin molecules on the protein cannot be controlled. This lack of positional control results in a heterogeneous mixture of proteins that attach to the solid surface in random orientations.

**Figure 5:**
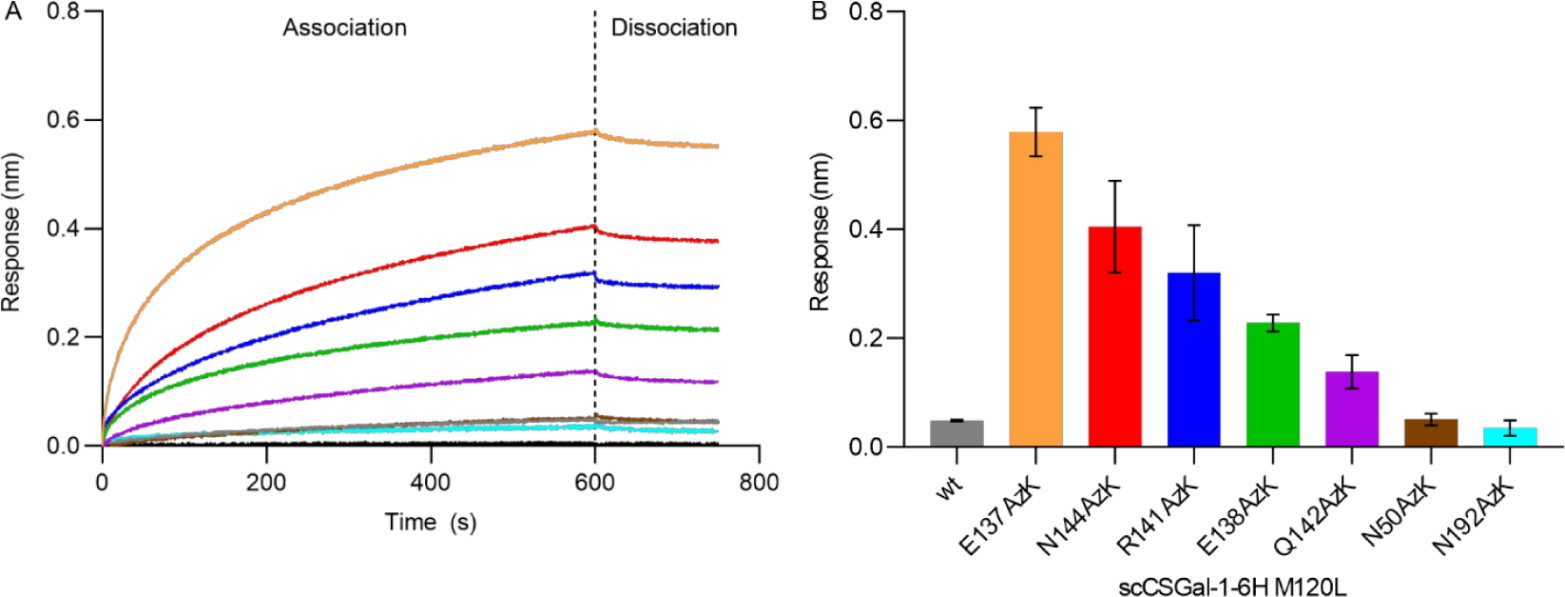
Interaction of vWF with scCSGal-1–6H M120L wt-ran-bio and scCSGal-1–6H M120L AzK-ss-bio variants. (A) Association and dissociation curves of vWF interaction with SA biosensor tips immobilized with scCSGal-1–6H M120L wt-ran-bio (gray) and scCSGal-1–6H M120L AzK-ss-bio variants: E137AzK (orange), E138AzK (light green), R141AzK (dark blue), Q142AzK (purple), N144AzK (red), N50AzK (brown), N192AzK (cyan). The black curve represents the sample diluent as a reference. vWF was applied at a constant concentration of 25 μg/mL. The curves present the mean values from triplicate measurements. Data show that oriented immobilization of scCSGal-1–6H M120L AzK-ss-bio variants enhanced binding sensitivity to the vWF compared to random immobilization of scCSGal-1–6H M120L wt-ran-bio. (B) Bars and error bars represent the mean Rt values and standard deviation from triplicate measurements of vWF association to surface immobilized with scCSGal-1–6H M120L AzK-ss-bio variants and scCSGal-1–6H M120L wt-ran-bio. The Rt of vWF interaction with the surface immobilized with scCSGal-1–6H M120L E137AzK-ss-bio was the highest.

To examine whether oriented immobilization of scCSGal-1–6H M120L AzK-ss-bio variants improved the sensitivity of binding to the target protein vWF compared to random immobilization of scCSGal-1–6H M120L wt-ran-bio, we employed the biosensor platform Bio-Layer Interferometry (BLI). A standard BLI assay includes four essential steps: equilibration of the biosensor tips in buffer, immobilization of the ligand on the biosensor tips, association of the analyte with the immobilized ligand, and dissociation of the analyte from the ligand. Here, the assay started with the equilibration of the SA biosensor tips in the sample diluent (as defined in the materials and methods section) to measure the baseline signal for the calculation of the LOQ. The LOQ is defined as the lowest concentration at which the analyte can be reliably detected, typically corresponding to a signal-to-noise ratio of 10:1.^66^ The baseline noise of the initial 60 s with the sample diluent was 0.0037 nm (*n* = 8). Therefore, the LOQ was calculated to be 0.037 nm. For the immobilization step, we optimized the concentration and loading performance of the scCSGal-1–6H M120L wt-ran-bio and scCSGal-1–6H M120L AzK-ss-bio variants. Ultimately, we loaded the SA biosensor tips with 60 μg/mL of biotinylated proteins, which resulted in a loading signal between 0.5 and 0.7 nm (Figure S7). Comparable response levels during the loading step indicated that similar amounts of all variant proteins were immobilized. Consequently, any differences in vWF binding could be attributed to the orientation of the proteins on the streptavidin biosensor tips rather than differences in protein amount. To assess nonspecific ligand binding, nonbiotinylated proteins, scCSGal-1–6H M120L wt and scCSGal-1–6H M120L N192AzK, were used. No nonspecific interactions of nonbiotinylated proteins with SA biosensor tips occurred, which confirmed the selectivity of the immobilization procedure (Figure S8). This finding further confirmed the successful biotinylation of the scCSGal-1–6H M120L wt and AzK variants, as only biotinylated proteins could be immobilized onto the SA surface. Subsequently, an additional equilibration step (120 s) with sample diluent was introduced to remove the excess of biotinylated scCSGal-1–6H M120L and to achieve a constant loading baseline. Afterward, the glycoprotein vWF was applied to the SA biosensor tips coated with scCSGal-1–6H M120L AzK-ss-bio and scCSGal-1–6H M120L wt-ran-bio to determine the corresponding association profiles and binding responses (Figure 5). For the association step, an optimized vWF concentration of 25 μg/mL was used to achieve a reliable signal. When 25 μg/mL of vWF was added to the surface of scCSGal-1–6H M120L wt-ran-bio, vWF binding generated only a weak response (Figure 5A, gray line). The specific response at 600 s (Rt, *t* = 600 s), calculated as the average of three independent measurements ± standard deviation, was 0.049 ± 0.0028 nm. On the other hand, when 25 μg/mL of vWF was applied to the surface of scCSGal-1–6H M120L AzK-ss-bio, binding of vWF was clearly observed (Figure 5A). As expected, scCSGal-1–6H M120L AzK-ss-bio variants with immobilization positions in the linker region demonstrated detectable and increased binding responses compared to the randomly immobilized scCSGal-1–6H M120L wt-ran-bio. The other two AzK variants (N50AzK and N192AzK), which we had chosen as alternative immobilization positions outside of the linker sequence, generated only weak response signals (Figure 5A). While N50AzK, with an Rt of 0.051 ± 0.011 nm, showed almost nondetectable binding, the Rt of N192AzK (0.035 ± 0.014 nm) was below the LOQ. Nevertheless, there was a clear difference in response between the AzK variants. The E137AzK variant exhibited the highest Rt value of about 0.58 ± 0.044 nm, which is about 12 times higher than the Rt generated by the scCSGal-1–6H M120L wt-ran-bio. The specific responses of other AzK variants were in the following order: 0.40 ± 0.084 nm for N144AzK, 0.32 ± 0.088 nm for R141AzK, 0.23 ± 0.016 nm for E138AzK, and 0.14 ± 0.031 nm for Q142AzK (Figure 5B). This variation in response among the five AzK variants is puzzling but may be explained as follows: Although these positions are located in the linker region and are close to one another, each position is surrounded by distinct amino acid residues forming unique local environments. Subtle changes in these environments may have long-distance effects on ligand binding.^67^ Neighboring residues can vary significantly in their properties such as charge or hydrophobicity, meaning that the substitution of individual amino acids by AzK can alter the local structural and/or biochemical context in the protein. For example, replacing a charged residue like E137, R141 or E138 with AzK in the linker region could disrupt local ionic interactions, thereby (subtly) altering the protein’s orientation, conformation and/or flexibility, which might lead to varied binding responses to vWF. This variability highlights the importance of systematically exploring multiple positions for ncAA incorporation to identify the optimal position for functional performance.

The reproducibility of the test performance was evaluated using vWF and immobilized scCSGal-1–6H M120L wt-ran-bio, as described above, with *n* = 8. The corresponding Rt was 0.049 nm, with a relative standard deviation (RSD) of 3.4%. The interaction of vWF with the SA surface was shown to be negligible, confirming that the SA itself has no cross-reactivity with vWF (Figure S8B).

**Figure 6:**
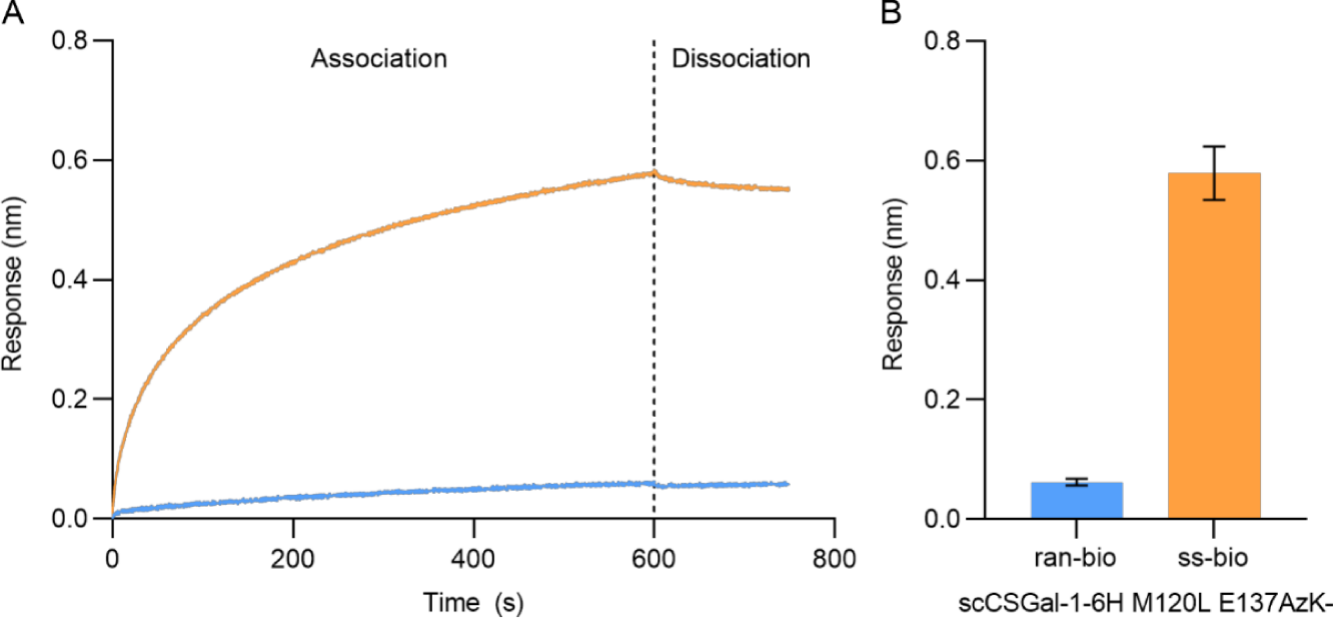
Interaction of vWF with scCSGal-1–6H M120L E137AzK-ss-bio and scCSGal-1–6H M120L E137AzK-ran-bio. (A) Association and dissociation curves of vWF for SA sensors immobilized with scCSGal-1–6H M120L E137AzK-ran-bio (light blue) and scCSGal-1–6H M120L E137AzK-ss-bio (orange). vWF was applied at a concentration of 25 μg/mL. The curves represent the mean values of three independent measurements. Oriented immobilization of scCSGal-1–6H M120L E137AzK-ss-bio demonstrated significantly increased binding efficiency compared to scCSGal-1–6H M120L E137AzK-ran-bio. (B) Bars and error bars represent the mean Rt values and standard deviation of triplicate measurements of vWF association to scCSGal-1–6H M120L E137AzK-ss-bio and scCSGal-1–6H M120L E137AzK-ran-bio immobilized surfaces, respectively. scCSGal-1–6H M120L E137AzK-ss-bio immobilized surface exhibited 10-fold higher Rt than randomly immobilized scCSGal-1–6H M120L E137AzK-ran-bio.

In addition, we randomly biotinylated the best-performing E137AzK variant (scCSGal-1–6H M120L E137AzK-ran-bio) using NHS ester conjugation and the NHS ester-PEG4-biotin linker as described above. To ensure purity and prevent potential interference from copurified *E. coli* host proteins, as explained for the wild-type protein, the scCSGal-1–6H M120L E137AzK variant intended for NHS ester coupling was also repurified using SEC. When 25 μg/mL of vWF was added to the SA surface immobilized with scCSGal-1–6H M120L E137AzK-ran-bio, we observed a weak response of 0.062 ± 0.0058 nm. This Rt was about 10-fold lower than that observed with the surface immobilized with scCSGal-1–6H M120L E137AzK-ss-bio (Figure 6A,B) and similar to the Rt observed with scCSGal-1–6H M120L wt-ran-bio (0.049 ± 0.0028 nm). This finding further confirmed that the increased binding sensitivity of scCSGal-1–6H M120L E137AzK-ss-bio resulted primarily from the immobilization strategy and not because of incorporated ncAA.

**Figure 7:**
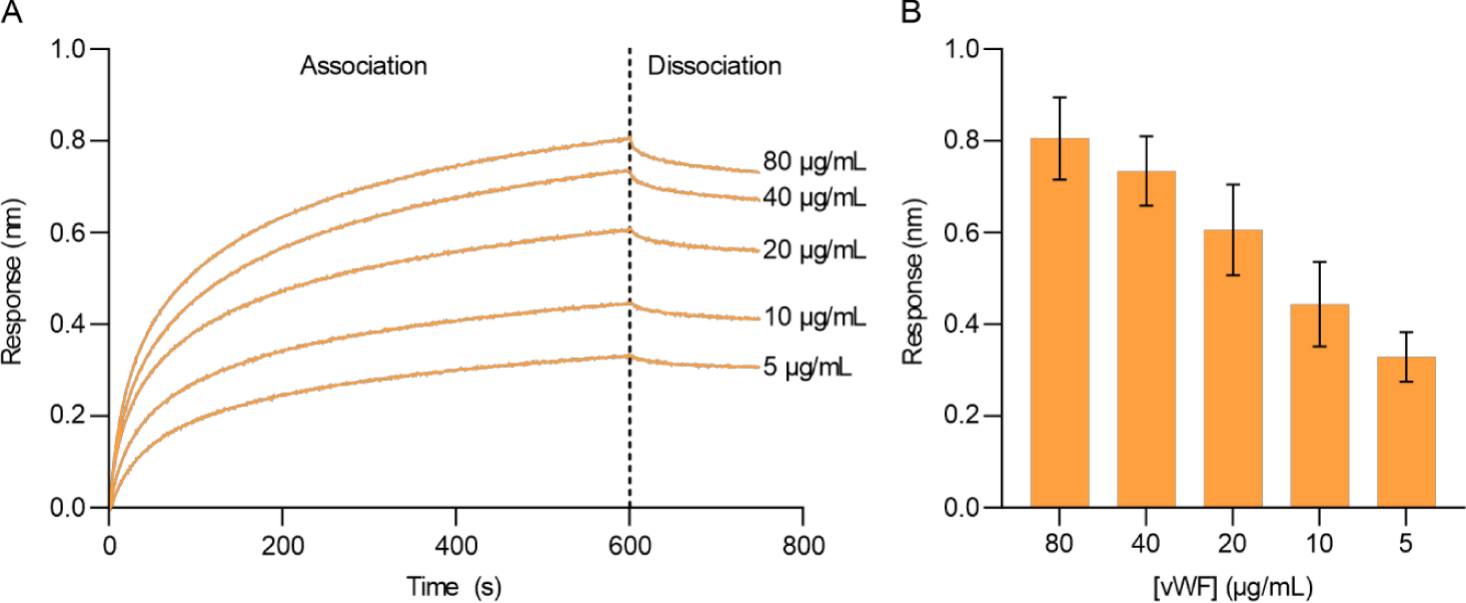
Concentration-dependent binding of scCSGal-1–6H M120L E137AzK-ss-bio with vWF. (A) Association and dissociation curves of vWF at concentrations of 5, 10, 20, 40, and 80 μg/mL. SA biosensor tips were immobilized with scCSGal-1–6H M120L E137AzK-ss-bio prior to vWF association. The curves represent the mean values from triplicate measurements at each concentration. (B) Bars and error bars represent the mean Rt values and standard deviation from triplicate measurements of vWF association at concentrations of 5, 10, 20, 40, and 80 μg/mL to the scCSGal-1–6H M120L E137AzK-ss-bio immobilized surface. scCSGal-1–6H M120L E137AzK-ss-bio interacts with vWF in a concentration-dependent manner.

To gain insight into the high binding efficiency of vWF to the scCSGal-1–6H M120L E137AzK-ss-bio immobilized surface, we aimed to estimate the apparent binding affinity of vWF to scCSGal-1–6H M120L E137AzK-ss-bio. Given that scCSGal-1–6H M120L contains two binding sites and vWF is a multimeric glycoprotein, the interaction between these molecules is rather complex. Therefore, the interferometry data cannot be fit using a simple 1:1 Langmuir binding model or a more complex binding model such as 2:1 or 1:2 binding. As a result, we were not able to obtain an apparent *K*_d_ of the interaction between vWF and the scCSGal-1–6H M120L E137AzK-ss-bio immobilized surface. To address this shortcoming, we explored the steady state analysis approach, which allows *K*_d_ determination without requiring curve fitting to a specific binding model. However, steady state analysis requires that the association binding curves reach equilibrium at each analyte concentration in the titration. We applied various concentrations of vWF (5–80 μg/mL) to the respective scCSGal-1–6H M120L E137AzK-ss-bio immobilized surface. Unfortunately, our experiments revealed that the binding curves did not reach equilibrium for every vWF concentration within the experimental time frame (Figure 7A). Consequently, it was not feasible to calculate the *K*_d_ using this method. However, the results demonstrated that vWF interacted with scCSGal-1–6H M120L E137AzK-ss-bio in a concentration-dependent manner (Figure 7A, B). Notably, oriented immobilization of scCSGal-1–6H M120L E137AzK-ss-bio enabled the detection of a reliable signal even at a vWF concentration as low as 5 μg/mL. In contrast, when we applied vWF at the same concentration range (5–80 μg/mL) to the scCSGal-1–6H M120L wt-ran-bio immobilized surface, no significant increase in response was observed. Even at a 2× higher vWF concentration of 160 μg/mL, the interaction did not result in a response greater than 0.06 nm (data not shown). Overall, these results demonstrated that the oriented immobilization of site-specifically biotinylated scCSGal-1–6H M120L E137AzK, achieved *via* AzK incorporation and click chemistry, significantly enhanced binding sensitivity compared to the immobilization of randomly biotinylated scCSGal-1–6H M120L wt through the conventional biotinylation method.

## 3 Conclusions

In this study, we successfully incorporated the ncAA AzK at strategically selected positions in scCSGal-1–6H M120L using the SCS method. By employing SPAAC, we achieved site-specific biotinylation and consequently controlled, oriented immobilization of scCSGal-1–6H M120L AzK-ss-bio variants on SA biosensor surfaces. BLI results revealed that this directed immobilization *via* ncAA and click chemistry significantly enhanced the binding sensitivity of scCSGal-1–6H M120L AzK-ss-bio variants to vWF compared to random immobilization using conventional NHS ester coupling. Specifically, the scCSGal-1–6H M120L E137AzK-ss-bio variant exhibited a 12-fold increase in sensitivity over the randomly immobilized wild-type protein. Further, the oriented immobilization of scCSGal-1–6H M120L E137AzK-ss-bio enabled reliable detection of vWF at concentrations as low as 5 μg/mL, which was not achievable with random immobilization. These findings underscore the importance of controlled protein orientation in biosensor design. They demonstrate that single site-specific genetic encoding of ncAAs combined with click chemistry could be a powerful approach for developing more sensitive and reliable diagnostic tools compared to the standard immobilization method.

Lectins, with their ease of recombinant production and cost-effectiveness compared to antibodies, offer a promising complement to antibodies in glycan detection.^68^ Future research may explore the expansion of the strategy described here to other lectins and immobilization platforms, potentially broadening the use of lectins as diagnostic tools. Additionally, vWF deficiency or dysfunction is associated with von Willebrand disease. The enhanced sensitivity of scCSGal-1–6H M120L E137AzK-ss-bio for detecting vWF could advance diagnostic assays and deepen our understanding of vWF-related pathologies.

## 4 Materials and Methods

### 4.1 Construction of Expression Plasmids

The expression plasmid pT7 × 31^43^ encoding scCSGal-1–6H M120L was constructed in the following way. To introduce a 6H-tag at the C-terminus, the *6H-scCSGal-1d3* gene was amplified by PCR using primers pBP2780 and pBP2781, with pT7 × 31_*6H-scCSGal-1d3* as the template (manuscript in preparation). The PCR product was resolved on a 0.8% (w/v) agarose gel and purified using the Wizard SV Gel and PCR Clean-Up kit (Promega Corporation, Madison, WI). Both the purified *scCSGal-1–6H* insert and the pT7 × 31 expression vector were digested with the restriction enzymes BglII/NdeI (Thermo Fisher Scientific Inc., Waltham, MA) and ligated using T4 DNA ligase (Thermo Fisher Scientific Inc.). Chemically competent *E. coli* Top10F′cells (Thermo Fisher Scientific Inc.) were heat-shock transformed with the ligation mixture, regenerated, and plated on Luria–Bertani (LB) agar plates containing 50 μg/mL kanamycin as described elsewhere.^69^ The resulting plasmid, pT7 × 31_*scCSGal-1–6H,* was isolated using the Wizard Plus SV Minipreps DNA Purification System (Promega Corporation) and the gene of interest was sequence verified (Microsynth AG, Balgach, Switzerland). The chemically competent *E. coli* BL21(DE3) expression strain (Merck KGaA, Darmstadt, Germany) was transformed with the sequence-verified pT7 × 31_*scCSGal-1–6H* plasmid and stored in 30% (v/v) glycerol at −80 °C.

To replace methionine at position 120 with leucine, the *scCSGal-1–6H M120L* insert was generated through overlap extension (OE) PCR. Two DNA fragments of 383 bp and 531 bp were produced in separate PCR reactions using primer pairs pBP2826/pBP2829 and pBP2828/pBP2827, respectively, with the pT7 × 31_*scCSGal-1–6H* plasmid as the template. These fragments were then spliced together *via* OE PCR using primers pBP2826 and pBP2827, resulting in the *scCSGal-1–6H M120L* insert. The spliced PCR product was resolved on a 0.8% (w/v) agarose gel, purified, digested with BglII/NdeI, and ligated into the BglII/NdeI-linearized pT7 × 31 vector to produce pT7 × 31_*scCSGal-1–6H M120L*. The procedure of transformation and sequence verification of this construct was performed as described above.

The analogous procedure was followed to prepare the *scCSGal-1–6H M120L* inserts with an amber (am) stop codon at positions E137, E138, R141, Q142, N144, N50, and N192. Overlap primers pBP2732/pBP2733, pBP2734/pBP2735, pBP2736/pBP2737, pBP2738/pBP2739, pBP2740/pBP2741, pBP2742/pBP2743, and pBP2744/pBP2745 were used in PCR reactions to generate each insert, followed by OE PCR with primers pBP2826 and pBP2827. Each insert was digested with BglII/NdeI and separately ligated into the BglII/NdeI-linearized pT7 × 31 vector, resulting in plasmids pT7 × 31_*scCSGal-1–6H M120L E137am*, pT7 × 31_*scCSGal-1–6H M120L E138am,* pT7 × 31_*scCSGal-1–6H M120L R141am,* pT7 × 31_*scCSGal-1–6H M120L Q142am,* pT7 × 31_*scCSGal-1–6H M120L N144am,* pT7 × 31_*scCSGal-1–6H M120L N50am, and* pT7 × 31_*scCSGal-1–6H M120L N192am*. Chemically competent *E. coli* TOP10F’ cells were transformed with the resulting plasmids. Plasmids from selected kanamycin-resistant clones were isolated and sequence verified. Subsequently, *E. coli* BL21(DE3) cells were transformed with the sequence-verified plasmids and stored as glycerol stocks at −80 °C. All primers used in this study were purchased from Integrated DNA Technologies, Inc., Coralville, IA and are listed in Table S1.

### 4.2 Protein Production

A volume of 10 mL LB medium containing 50 μg/mL kanamycin (LBKan) was inoculated with the corresponding glycerol stocks and incubated overnight at 37 °C with vigorous shaking. The overnight culture (ONC) was then added to 350 mL of fresh LBKan medium at a starting attenuance (*D*_600_) of 0.1. The cultures were incubated at 37 °C with shaking at 130 rpm until they reached a *D*_600_ of 0.8. Gene expression was induced with 1 mM IPTG, and the cultures were incubated at 20 °C for 18 h with shaking at 130 rpm. To produce the azide-labeled variant lectins, the noncanonical amino acid H-l-Lys(EO-N3)-OH·HCl (AzK; Iris Biotech, Marktredwitz, Germany) was freshly prepared in sterile doubly distilled H_2_O (ddH_2_O) and added to the cultures at a final concentration of 5 mM at the time of IPTG induction.

To assess the expression of the AzK variants, a volume of the cell culture equivalent to a D_600_ of 0.8 was collected 18 h after IPTG induction and AzK addition. After harvesting the cells by centrifugation at 17000 *g* for 2 min, the medium supernatant was discarded, and the cell pellets were resuspended in 80 μL of SDS sample buffer (50 mM Tris, 4% (v/v) glycerol, 1% SDS, 100 mM dithiothreitol, 0.04% (w/v) bromophenol blue, 0.5% (v/v) 2-mercaptoethanol). The samples were heated at 95 °C for 10 min. Insoluble debris was sedimented by centrifugation at 17000 *g* for 5 min, and the supernatant was analyzed by SDS-PAGE. The remaining culture volume was harvested by centrifugation at 8000 rpm (JA-10 rotor, Beckman Coulter Life Sciences, Indianapolis, IN) for 10 min, and the pellets were stored at −20 °C until further processing.

### 4.3 Protein Purification

#### 4.3.1 Immobilized Metal Affinity Chromatography (IMAC)

Thawed cell pellets were resuspended in 25 mL of lysis buffer (20 mM Tris, 300 mM NaCl, 10 mM imidazole, pH 8). The cells were lysed by sonication using a Branson Sonifier 250 (Emerson Electric, St. Louis, MO) for 3 × 1 min, with 1 min of cooling on ice between each interval. The lysate was centrifuged at 20000 rpm (JA-25.50 rotor, Beckman Coulter Life Sciences, Indianapolis, IN) for 30 min at 4 °C.

The clarified cell lysate was loaded onto Zn^2+^-charged sepharose beads (Chelating Sepharose Fast Flow, Cytiva, Marlborough, MA) that had been previously equilibrated with lysis buffer. The flow-through was collected by gravity and nonspecifically bound proteins were removed with wash buffer (20 mM Tris, 300 mM NaCl, 20 mM imidazole, pH 8). The target lectins were eluted using an elution buffer consisting of 20 mM Tris, 300 mM NaCl and 300 mM imidazole at pH 8. Aliquots of the elution fractions were mixed with SDS sample buffer for SDS-PAGE analysis. Elution fractions with the highest concentration and purity of the target proteins, as determined by SDS-PAGE, were pooled, and the buffer was exchanged to phosphate-buffered saline (PBS; 1.44 g/L Na_2_HPO_4_, 8.0 g/L NaCl, 0.2 g/L KCl and 0.24 g/L KH_2_PO_4_, pH 7.4) using PD-10 desalting columns (Cytiva). The protein concentrations of scCSGal-1–6H M120L wt and the AzK variants were determined using a NanoDrop 2000 spectrophotometer (Thermo Fisher Scientific Inc.), applying an extinction coefficient of 16.96 × 10^3^ M^–1^ cm^–1^ and calculated molecular weight of 30.9 kDa. For storage, small aliquots of the protein solutions were lyophilized and kept at 4 °C until further use.

#### 4.3.2 Lactose Affinity Chromatography

The purification by lactose affinity chromatography was performed as described by Tobola et al.^32^ In short, proteins were loaded onto a lactose–agarose column (Lactose Separopore 6B-CL; BioWorld, Dublin, OH) and nonspecifically bound proteins were removed by washing with 0.1 M sodium phosphate (NaPi) buffer containing 150 mM NaCl, pH 7.2. The column-bound proteins were eluted using the same buffer supplemented with 0.1 M α-lactose (Carl Roth GmbH, Karlsruhe, Germany). For SDS-PAGE analysis, each protein sample was mixed with SDS sample buffer and heated at 95 °C for 10 min. After elution, the fractions were pooled, and the buffer was exchanged to PBS, pH 7.4, using PD-10 desalting columns. Protein concentrations were determined spectrophotometrically. The purified protein solutions were aliquoted, lyophilized, and stored at 4 °C until further use.

### 4.4 Conjugation Reaction with Fluorophore

Lactose-purified proteins, scCSGal-1–6H M120L wt and its AzK variants, were individually conjugated with a 10-fold molar excess of the fluorophore dibenzocyclooctyne-sulfo-Cy3 (DBCO-Cy3; Jena Bioscience GmbH, Jena, Germany). The reaction mixtures were incubated at room temperature with shaking at 600 rpm for 2 h in the dark to facilitate SPAAC. The reaction was stopped by adding SDS sample buffer, followed by heating the samples at 95 °C for 10 min.

### 4.5 Sodium Dodecyl Sulfate Polyacrylamide Gel Electrophoresis

Protein samples were separated on 4–12% polyacrylamide (PA) gels (NuPAGE bis-Tris mini protein precast gels, Invitrogen, Waltham, MA). SDS-PA gels were run at 200 V for 40 min using NuPAGE MES-SDS running buffer (Thermo Fisher Scientific Inc.). The gels were stained with InstantBlue Coomassie Protein Stain (Abcam plc., Cambridge, UK).

The gels containing fluorophore-conjugated proteins were washed three times for 15 min each to remove the unbound fluorophore. The gel was visualized under UV illumination at 302 nm before being stained with InstantBlue Coomassie Protein stain to confirm protein presence and integrity.

### 4.6 Biotinylation

The linkers DBCO-PEG4-biotin and NHS ester-PEG4-biotin from Broadpharm (San Diego, CA) were dissolved in ddH_2_O to a concentration of 33.3 mM and 16.9 mM, respectively. Site-specific (ss) biotinylation of IMAC-purified scCSGal-1–6H M120L AzK variants was carried out using SPAAC with the DBCO-PEG4-biotin linker. The reaction was prepared in a total volume of 130 μL PBS, pH 7.4, containing 15 μM of the AzK variant and 300 μM DBCO-PEG4-biotin, and incubated overnight at room temperature in the dark. For random (ran) biotinylation of scCSGal-1–6H M120L wt and scCSGal-1–6H M120L E137AzK, the NHS ester-PEG4-biotin linker was used. Briefly, the reaction was prepared in 130 μL of PBS pH 7.4 containing 20 μM protein and 400 μM biotin linker and incubated overnight at 4 °C in the dark. All reaction mixtures were desalted using Zeba Spin desalting columns (7 kDa molecular weight cutoff (MWCO), 0.5 mL, Thermo Fisher Scientific Inc.) with PBS, pH 7.4, to remove excess of nonreacted free biotin reagent. The concentrations of scCSGal-1–6H AzK-ss-bio and scCSGal-1–6H wt-ran-bio were measured using a NanoDrop spectrophotometer as described previously.

### 4.7 Bio-Layer Interferometry (BLI)

BLI studies were performed using the Octet RED96e instrument (ForteBio, Fremont, CA) with black 96-well plates (Nunc F96 MicroWell Black Polystyrene Plate, Thermo Fisher Scientific Inc.). Biotinylated ligands, scCSGal-1–6H M120L wt and its azido variants, as well as the analyte vWF (Sino Biological, Inc., Beijing, China), were diluted in PBS containing 0.05% Tween 20 and 0.1% BSA, pH 7.4 (referred to as the sample diluent). Each sample was prepared in a total working volume of 200 μL per well, and all assay steps equilibration, loading, association, and dissociation were carried out at 25 °C with shaking at 1000 rpm.

Prior to each assay, SA biosensor tips (ForteBio) were prewetted in 200 μL of sample diluent for at least 10 min, followed by equilibration with fresh sample diluent for 60 s. The SA biosensor tips were then loaded with biotinylated scCSGal-1–6H M120L AzK-ss-bio variants or scCSGal-1–6H M120L wt-ran-bio at a concentration of 60 μg/mL, followed by an additional 120 s equilibration step with sample diluent. For the association phase, vWF at a concentration of 25 μg/mL was introduced to the biosensor tips immobilized with either scCSGal-1–6H M120L AzK-ss-bio or scCSGal-1–6H M120L wt-ran-bio. Additionally, vWF was tested at concentrations of 5, 10, 20, 40, and 80 μg/mL for binding to the scCSGal-1–6H M120L E137AzK-ss-bio immobilized surface. Association at each concentration was monitored for 600 s, followed by a dissociation phase in sample diluent for 150 s. All measurements were performed in triplicate.

### 4.8 BLI Data Analysis

Raw data generated with Octet Software (Version 11.0, ForteBio) were exported to Excel spreadsheets (Version 2003, Microsoft, Redmond, WA). The raw data of the vWF association and dissociation responses were aligned to the individual association step and plotted versus time using GraphPad Prism software (version 9.5.1, GraphPad Software, Boston, MA). The specific response R at a defined time t (Rt, *t* = 600 s) for each sample was calculated as the average of three independent measurements ± standard deviation (SD) using GraphPad Prism software. The reproducibility of Rt was determined with *n* = 8, and the relative standard deviation (RSD) was calculated by dividing the standard deviation by the mean and multiplying by 100. The limit of quantitation (LOQ) for vWF association was estimated by determining the minimum concentration at which the analyte could be reliably quantified, typically corresponding to a signal-to-noise ratio of 10:1. Baseline noise was measured during the initial 60 s sample diluent step (*n* = 8).

## Supporting Information Available

Additional materials and methods; Figure S1: chemical structure of AzK; Figure S2: SDS-PA gel after purification of scCSGal-1–6H; Figure S3: SDS-PA gel after purification of scCSGal-1–6H M120L; Figure S4: SDS-PA gel after purification of scCSGal-1–6H M120L wt and AzK variants; Figure S5: ESI-MS spectra of scCSGal-1–6H M120L wt and AzK variants; Figure S6: DSF plots of scCSGal-1–6H M120L wt and AzK variants; Figure S7: loading traces of scCSGal-1–6H M120L wt-ran-bio and AzK-ss-bio variants from BLI measurements; Figure S8: controls for BLI measurements; Table S1: DNA and amino acid sequences; Table S2: ESI-MS data analysis; Table S3: apparent binding affinities of scCSGal-1–6H M120L wt and its AzK variants from DSF data (PDF)
