# Supplementary material for: Site-Specific Immobilization Boosts the Performance of a Galectin-1 Biosensor
Source: Bioconjug Chem. 2024 Dec 3;35(12):1944–58. doi: 10.1021/acs.bioconjchem.4c00467 (PMC11660155; doi:10.1021/acs.bioconjchem.4c00467)
Supplement: Supplementary file 1 — bc4c00467_si_001.pdf [file bc4c00467_si_001.pdf]

Supporting Information

Site-specific Immobilization Boosts the Performance of a Galectin-1 Biosensor

Dajana Kolanovic[a,b,1], Rajeev Pasupuleti[a,b], Jakob Wallner[c], Georg Mlynek[c,2], Birgit Wiltschi[a,d,*]

[a] acib – Austrian Centre of Industrial Biotechnology, Graz 8010, Austria

[b] Institute of Molecular Biotechnology, Graz University of Technology, Graz 8010, Austria

[c] BOKU Core Facility Biomolecular & Cellular Analysis, BOKU University, Vienna 1190, Austria

[d] Institute of Bioprocess Science and Engineering, Department of Biotechnology, BOKU University, Vienna 1190, Austria

[1] Present address: Austrian Institute of Technology GmbH, Center for Health and Bioresources, Competence Unit Molecular Diagnostics, Giefinggasse 4, 1210 Vienna, Austria

[2] Present address: Department of Structural and Computational Biology, Max Perutz Labs, University of Vienna, Campus Vienna Biocenter 5, A-1030 Vienna, Austria

[*]Corresponding author.

E-mail address: birgit.wiltschi@acib.at (B. Wiltschi)

Supporting Methods

Mass spectrometry analysis of intact proteins

Lyophilized proteins from purification by lactose-affinity chromatography (scCSGal-1-6H M120L wt and seven AzK variants) were reconstituted in ddH_2O and desalted immediately prior to analysis using Zeba™ Micro Spin desalting columns (7 kDa MWCO, 75 µL, Thermo Fisher Scientific Inc.). For liquid chromatography-electrospray ionization-mass spectrometry (LC-ESI-MS) analysis, 2 µg of each desalted protein was injected into a LC-ESI-MS system (LC: 1290 Infinity II UPLC, Agilent, Santa Clara, CA). A gradient elution from 15 to 80% acetonitrile in 0.1% (v/v) formic acid was applied over 15 min, using a Waters BioResolve column (2.1 x 5 mm) at a flow rate of 400 µL/min. Detection was performed with a quadrupole time-of-flight (Q-TOF) instrument (Series 6560 LC-IMS-QTOFMS, Agilent), equipped with a Jetstream ESI source operating in positive ion MS mode (range: 100-3200 Da). Instrument calibration was conducted with an ESI calibration mixture (Agilent). Data was processed using MassHunter BioConfirm B.08.00 software (Agilent), and the spectrum was deconvoluted by MaxEnt.

Differential scanning fluorimetry

Lyophilized protein samples obtained from IMAC purification were reconstituted with ddH_2O. To avoid any buffer mismatch in further measurements, the buffer for all protein samples was exchanged to PBS, pH 7.4 using Zeba™ Spin desalting columns (7 kDa MWCO, 0.5 mL, Thermo Fisher Scientific Inc.). The ligand lactose was also prepared in PBS, pH 7.4 at a stock concentration of 200 mM. Thermal shift assays were performed using 5 µM of protein in PBS pH 7.4, with final lactose concentrations ranging from 0 to 160 mM and 4x SYPRO Orange protein gel stain (Thermo Fisher Scientific Inc.) in a total reaction volume of 25 µl. Measurements were performed using a CFX Opus 96, Real-Time PCR System (Bio-Rad Laboratories, Inc., Hercules, CA). The temperature was gradually increased from

15 °C to 95 °C in 0.5 °C increments within an interval of 30 s, and fluorescence readings were recorded at each interval in FRET scanning mode. Measurements for each protein were performed in technical triplicates (n = 3). The resulting melting temperatures were exported using CFX Maestro software version 1.1 (Bio-Rad Laboratories, Inc.) and analyzed with the Foldaffinity webserver (https://spc.embl-hamburg.de/app/foldAffinity) to determine apparent binding affinities ($K_{d,\ app}$). The same procedure was used for the negative control with sucrose, though without replicates.

Supporting Figures & Tables

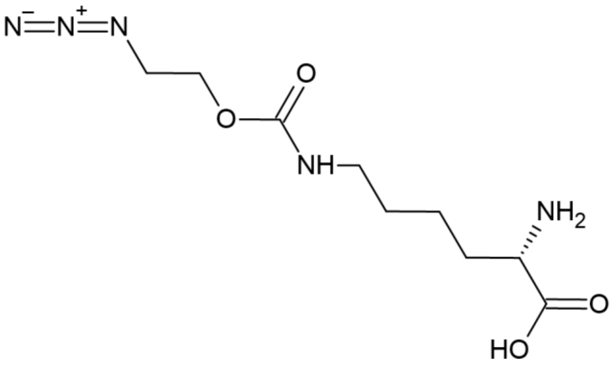

Figure S1. Chemical structure of ncAA AzK (N^{ε}-((2-azidoethoxy)carbonyl)-L-lysine).

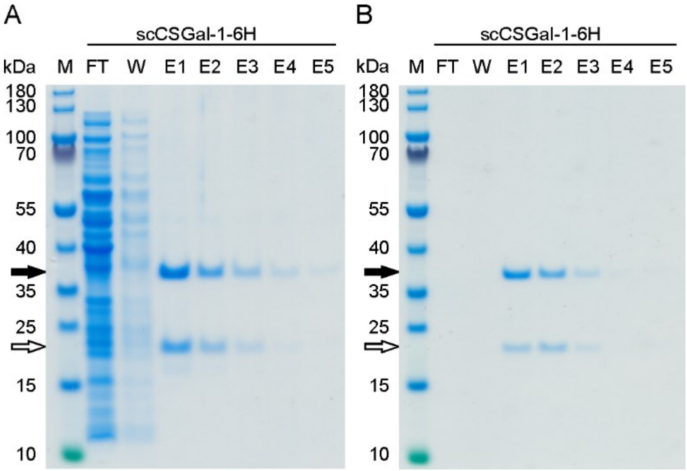

Figure S2. Purification of scCSGal-1-6H using lactose-affinity chromatography followed by Zn-IMAC. (A) Lactose-purified protein was analyzed on a 4-12% SDS-PA gel. scCSGal-1-6H remained functional despite the addition of the C-terminal 6H-tag, as evidenced by its efficient binding to the lactose column followed by elution. The Coomassie stained SDS-PA gel also shows an additional protein band above 15 kDa, indicating truncated but functional protein. (B) To explore the composition of the truncated protein, the lactose-purified protein sample was re-purified by zinc affinity chromatography. The analysis demonstrated that the truncated protein co-purified with the full-length protein, which confirmed the presence of a C-terminal 6H-tag in the truncated protein. The truncated protein migrated at >15 kDa, indicating that it consisted of approximately half of the scCSGal-1-6H. Lanes M, molecular size marker; FT, flowthrough; W, column wash; E1-E5, eluates 1-5. The numbers on the left margin of the gel indicate the size of the molecular weight marker bands in kDa. The black arrows indicate scCSGal-1-6H (MW_{calc} = 30.9 kDa) while white arrows indicate the truncated protein.

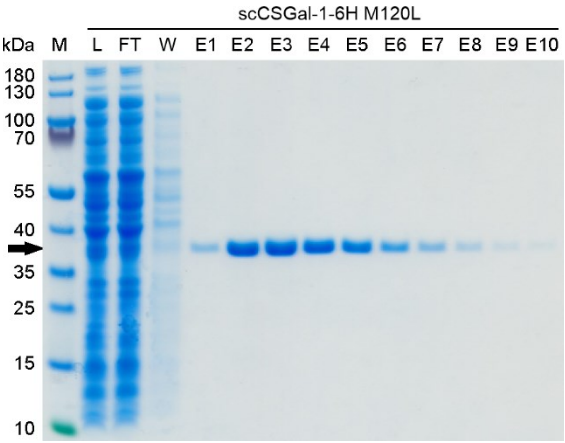

Figure S3. Purification of scCSGal-1-6H M120L using lactose-affinity chromatography. The SDS gel demonstrates that the replacement of M by L at position 120 resulted in the purification of only full-length scCSGal-1-6H M120L protein. This analysis also confirmed that the M120L mutation did not affect the protein's functionality, as evidenced by its binding to the lactose affinity column. Lanes M, molecular size marker; L, clarified lysate; FT, flowthrough; W, column wash; E1-E10, eluates 1-10. The numbers on the left margin of the gel indicate the size of the molecular weight marker bands in kDa. The MW_{calc} of scCSGal-1-6H M120L (black arrow) is 30.9 kDa. The 4-12% SDS-PA gel was stained with Coomassie protein stain.

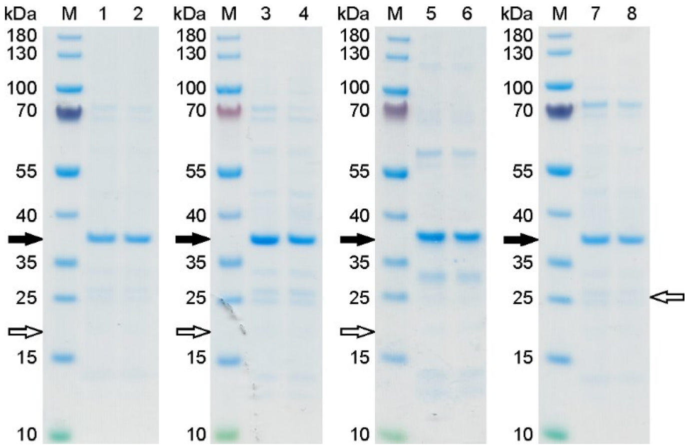

Figure S4. Purification of scCSGal-1-6H M120L wt and its AzK variants using Zn-IMAC to avoid interference from truncated proteins. Elution fractions were analyzed on a 4-12% SDS-PA gel. As expected, only full-length proteins were purified by Zn-IMAC (black arrows, lanes 2-8; MW_{calc} ~ 31 kDa). Truncated proteins resulting from un-suppressed amber codons could not be purified by Zn-IMAC due to the absence of a C-terminal 6H-tag. Consequently, truncated protein bands are not observed after Zn-IMAC purification (white arrows, lanes 2-8). Additional bands represent accidentally co-purified *E. coli* proteins. The white arrows indicate the regions where truncated variants, if present, are supposed to appear. Lane M, molecular size marker; lane 1, scCSGal-1-6H M120L wt; lanes 2-8, scCSGal-1-6H M120L variants: E137AzK (lane 2), E138AzK (lane 3), R141AzK (lane 4), Q142AzK (lane 5), N144AzK (lane 6), N50AzK (lane 7), and N192AzK (lane 8). The numbers on the left margin of the gels represent the size of the molecular weight marker bands in kDa.

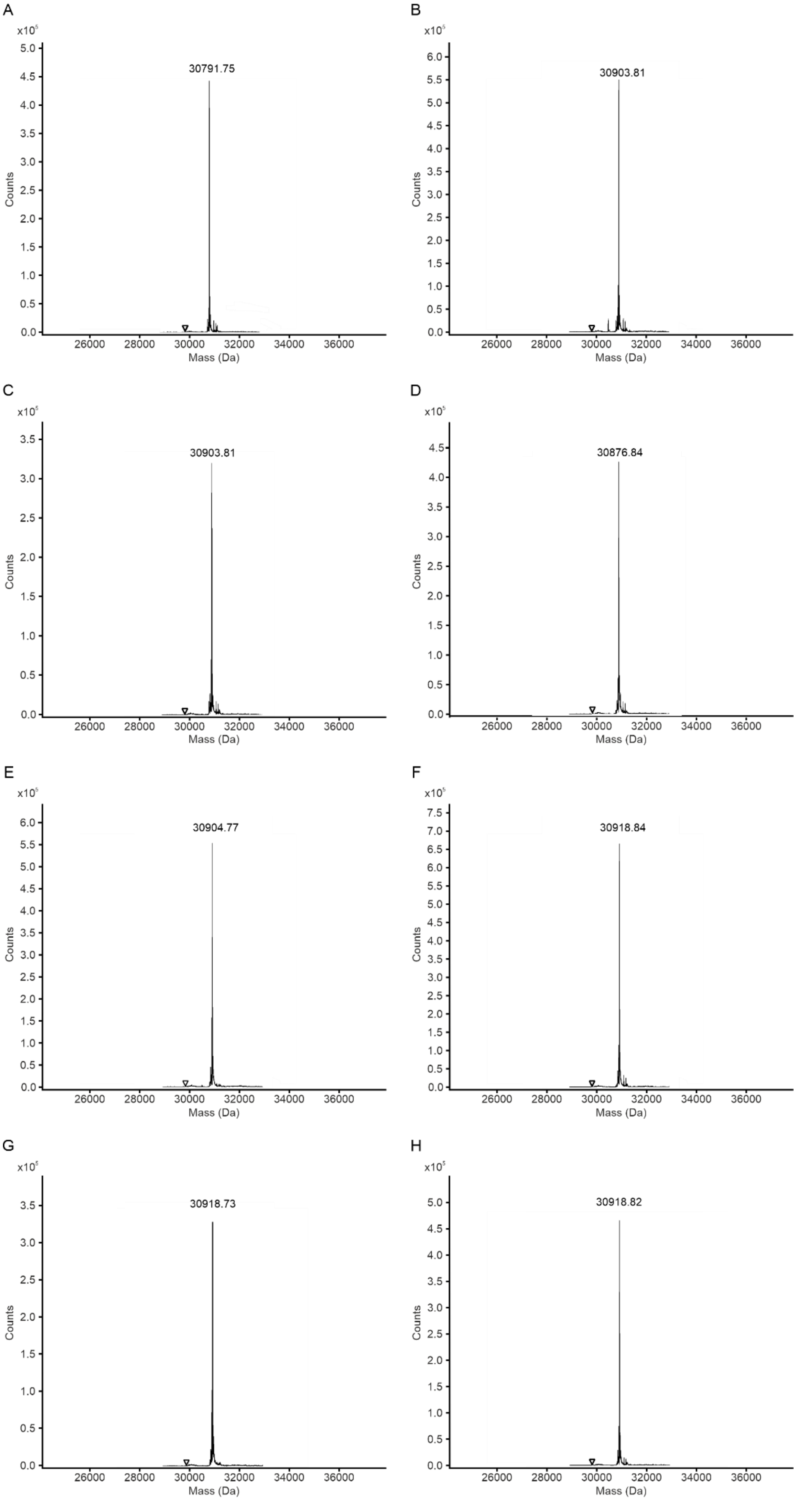

Figure S5. AzK incorporation in the scCSGal-1-6H M120L azido variants was confirmed by intact mass analysis. Electrospray ionization mass spectrometry (ESI-MS)

spectra of intact (A) scCSGal-1-6H M120L wt, (B) scCSGal-1-6H M120L E137AzK, (C) scCSGal-1-6H M120L E138AzK, (D) scCSGal-1-6H M120L R141AzK, (E) scCSGal-1-6H M120L Q142AzK, (F) scCSGal-1-6H M120L N144AzK, (G) scCSGal-1-6H M120L N50AzK, (H) scCSGal-1-6H M120L N192AzK. The proteins identified from the peaks are detailed in Table S2.

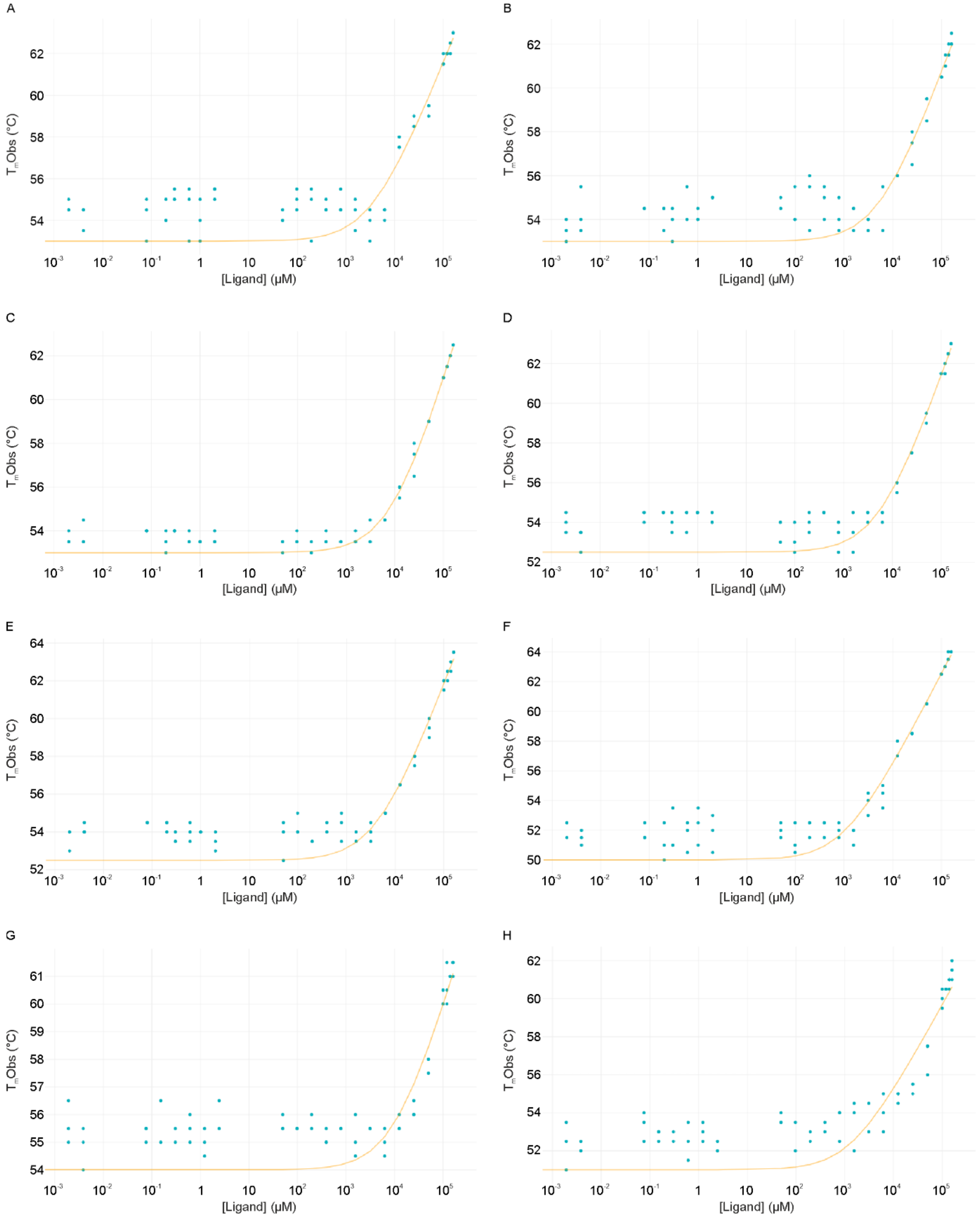

Figure S6. Confirmation of functionality and determination of apparent binding affinities ($K_{d,\ app}$) of scCSGal-1-6H M120L wt and AzK variants by fitting DSF data. Melting temperatures (T_ms) were estimated using the first derivative of DSF traces at different concentrations of the ligand lactose. Measurements were performed in technical triplicates (n = 3). Plots of (A), scCSGal-1-6H M120L wt; (B), scCSGal-1-6H M120L E137AzK; (C), scCSGal-1-6H M120L E138AzK; (D), scCSGal-1-

6H M120L R141AzK; (E), scCSGal-1-6H M120L Q142AzK; (F), scCSGal-1-6H M120L N144AzK; (G), scCSGal-1-6H M120L N50AzK; (H), scCSGal-1-6H M120L N192AzK were created by FoldAffinity (© eSPC, spc.embl-hamburg.de) using the “Tm fitting” model. scCSGal-1-6H M120L wt and AzK variants exhibited an increase in T_m with rising lactose concentrations. The stabilization of the proteins in the presence of lactose indicates successful ligand binding. Estimated $K_{d,\ app}$ values are listed in Table S3.

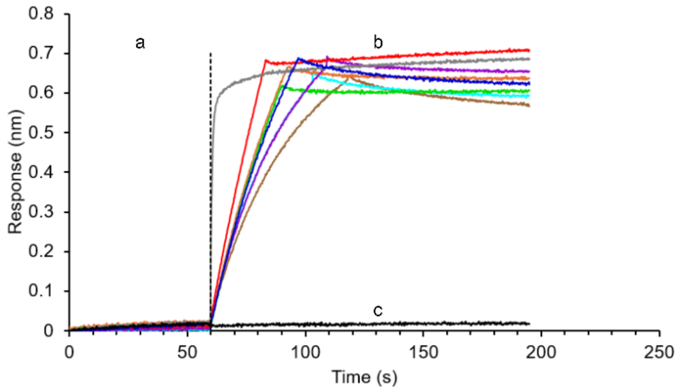

Figure S7. Loading traces of scCSGal-1-6H M120L wt-ran-bio and scCSGal-1-6H M120L AzK-ss-bio variants on SA biosensor tips. The plot shows (a) the equilibration step (60 s) with sample diluent, (b) the loading step with scCSGal-1-6H M120L wt-ran-bio (gray) and the scCSGal-1-6H M120L AzK-ss-bio variants: E137AzK (orange), E138AzK (light green), R141AzK (dark blue), Q142AzK (purple), N144AzK (red), N50AzK (brown), N192AzK (cyan). (c) Reference curve (black) showing equilibration and loading with sample diluent only.

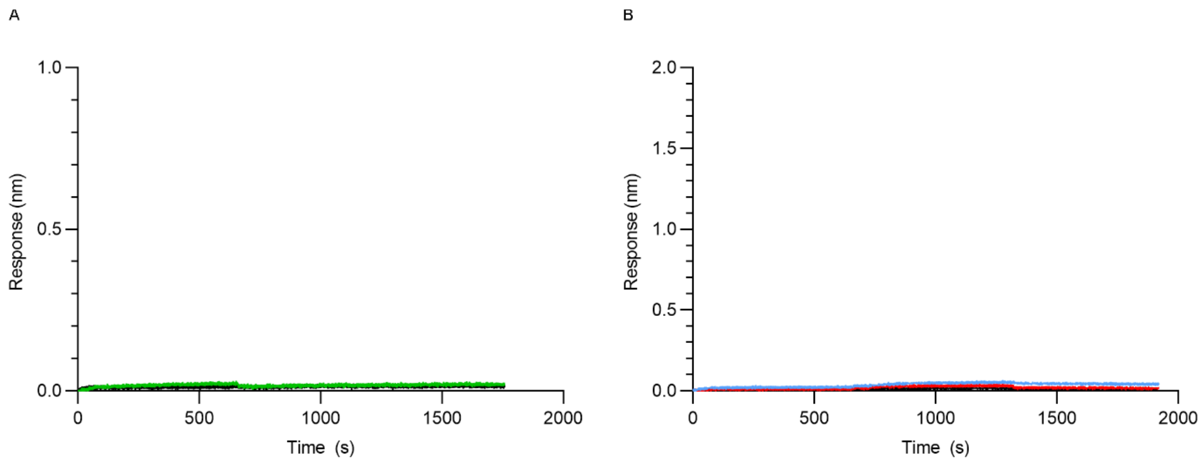

Figure S8. Evaluation of non-specific interactions of both the ligand and the analyte with SA biosensor tips. Non-specific ligand binding was assessed using non-biotinylated (A) scCSGal-1-6H M120L wt (light green) and (B) scCSGal-1-6H M120L N192AzK variant (red). No non-specific interaction of non-biotinylated lectins with the SA biosensor tips was observed. (B) vWF was applied to SA biosensor tips, and results demonstrated that non-specific interaction of vWF with the SA surface did not occur (light blue). The black curve represents the sample diluent as a reference.

Table S1. DNA and amino acid sequences in this study.

Name	Type	DNA sequence (5’ → 3’)
scCSGal-1-6H M120L	gene[a], [b],	ATCGCACATATGGCATCAGGCCTCGTCGCATCGAACCTGAACCT<br>TAAACCTGGCGAGAGCTTACGGGTTCGTGGGGAAGTAGCCCCTG<br>ATGCGAAATCATTTGTCTTAAACTTAGGCAAAGACTCTAACAAT<br>TTGTCACTCCATTTCAATCCTCGCTTCAATGCTCATGGGGACGC<br>AAACACCATCGTAAGCAACTCGAAGGACGGCGGTGCGTGGGGGA<br>CGGAACAACGCGAAGCGGTGTTTCCATTTCAACCAGGGAGCGTC<br>GCCGAGGTATCGATTACTTTTGACCAAGCAAATCTGACAGTAAA<br>ATTACCTGACGGTTACGAGTTCAAGTTTCCAAATCGCTTAAATC<br>T**GGAGG**CTATTAATTATCTGGCAGCTGATGGTGACTTCAAAATC<br>AAAAGCGTGGGTATTGACGGGACTGAAGAAGGGATGCGGCAAGG<br>TAACTCGCTTGTTGCATCAAATCTTAACTTGAAACCGGGGGAGT<br>CACTCCGCGTACGCGGCGAGGTTGCTCCGGATGCCAAGTCTTTC<br>GTGCTGAATCTGGGTAAGGATAGCAATAATCTCTCTTTGCATTT<br>TAATCCTCGGTTTAATGCACACGGTGACGCCAACACCATTGTCA<br>GCAATTCAAAGGATGGTGGTGCATGGGGTACAGAGCAACGGGAG<br>GCGGTCTTTCCGTTCCAACCAGGTTCAGTGGCGGAAGTCAGTAT<br>CACTTTCGACCAGGCGAATCTTACTGTAAAGCTCCCAGACGGTT<br>ATGAGTTCAAGTTCCCTAACCGTCTTAACCTCGAGGCTATTAAC<br>TATATGGCTGCCGATGGGGATTTTAAAATCAAGAGTGTAGCCTT<br>CGATGGCAGTCATCACCATCATCACCATTGATAAAGATCTATCG<br>TC
pBP2780_fw	primer[a],	ATCGCACATATGGCATCAGGCCTCGTCGCATCG
pBP2781_rev	primer[a],	GACGATAGATCTTTATCAATGGTGATGATGGTGATGACTGCCAT<br>CGAAGGCTACACTCTTGATTTTAAAATCCCC
pBP2826_fw	primer[a],	ATCGCACATATGGCATCAGGCCTCGTCG
pBP2827_rev	primer[a],	GACGATAGATCTTTATCAATGGTGATGATGGTGATGACTGCC
pBP2828_fw	primer	CTGGAGGCTATTAATTATCTGGCAGCTGATGG
pBP2829_rev	primer	CCATCAGCTGCCAGATAATTAATAGCCTCCAG
pBP2732_fw	primer	TGACGGGACTTAGGAAGGGATGCGGC
pBP2733_rev	primer	GCCGCATCCCTTCCTAAGTCCCGTCAAT
pBP2734_fw	primer	CGGGACTGAATAGGGGATGCGGCAAGG
pBP2735_rev	primer	GCCGCATCCCCTATTCAGTCCCGTCA
pBP2736_fw	primer	GACTGAAGAAGGGATGTAGCAAGGTAACTCGC
pBP2737_rev	primer	GCGAGTTACCTTGCTACATCCCTTCTTCAGTC
pBP2738_fw	primer	AGGGATGCGGTAGGGTAACTCGCTTG
pBP2739_rev	primer	GCGAGTTACCCTACCGCATCCCTTCTTCAGTCC
pBP2740_fw	primer	GATGCGGCAAGGTTAGTCGCTTGTTGC
pBP2741_rev	primer	GCAACAAGCGACTAACCTTGCCGCATCCC
pBP2742_fw	primer	TCCTCGCTTCTAGGCTCATGGGGACGC
pBP2743_rev	primer	GCGTCCCCATGAGCCTAGAAGCGAGGA
pBP2744_fw	primer	TCCTCGGTTTTAGGCACACGGTGACGC
pBP2745_rev	primer	GCGTCACCGTGTGCCTAAAACCGAGGAT

Table S1 continued

Name	Type	Amino acids sequence (N-terminus → C-terminus)
scCSGal-1-6H M120L	protein[c],[d],[e],[f],[g]	MASGLVASNLNLKPGESLRVRGEVAPDAKSFVLNLGKDSNNLSL HFNPRFNAHGDANTIVSNSKDGGAWGTEQREAVFPFQPGSVAEV SITFDQANLTVKLPDGYEFKFPNRLNLEAINYLAADGDFKIKSV GIDGTEEGMRQGNSLVASNLNLKPGESLRVRGEVAPDAKSFVLN LGKDSNNLSLHFNPRFNAHGDANTIVSNSKDGGAWGTEQREAVF PFQPGSVAEVSITFDQANLTVKLPDGYEFKFPNRLNLEAINYMA ADGDFKIKSVAFDGSHHHHHH

[a] Restriction sites are underlined.

[b] The nucleotides shown in blue were exchanged against the amber stop codon (TAG) for the site-specific incorporation of AzK; the sequence in bold indicates the internal Shine-Dalgarno sequence.

[c] The introduced hexahistidine-tag at the C-terminus, including a glycine-serine linker, is shown in brown.

[d] The leucine highlighted in red replaced methionine at position 120.

[e] The loop that was designed to connect two subunits is highlighted in light gray.

[f] Amino acid residues in the glycan binding domain are shown in magenta.

[g] The amino acids shown in cyan (E137), light pink (E378), red (R141), blue (Q142), green (N144), and dark green (N50, N192) are the sites for AzK incorporation in scCSGal-1-6H M120L.

Table S2. ESI-MS data analysis. The average mass calculated (m_{calc}) using massXpert[1] with N-terminal methionine excision and ionization level 1 $[M+H]^+$ was compared to the observed mass (m_{obs}) of the protein samples. The difference in mass (Δm) was derived from subtracting observed mass (m_{obs}) from calculated mass (m_{calc}) in Daltons (Da).

**Protein species**	**m_{calc} (Da)**	**m_{obs} (Da)**	**Δm (Da)**
scCSGal-1-6H M120L wt	30791.83	30791.75	0.08
scCSGal-1-6H M120L E137AzK	30903.96	30903.81	0.15
scCSGal-1-6H M120L E138AzK	30903.96	30903.81	0.15
scCSGal-1-6H M120L R141AzK	30876.89	30876.84	0.05
scCSGal-1-6H M120L Q142AzK	30904.94	30904.77	0.17
scCSGal-1-6H M120L N144AzK	30918.97	30918.84	0.13
scCSGal-1-6H M120L N50AzK	30918.97	30918.73	0.24
scCSGal-1-6H M120L N192AzK	30918.97	30918.82	0.15

Table S3. Apparent binding affinities ($K_{d,\,app}$) of scCSGal-1-6H M120L wt and the AzK variants for lactose, as assessed from DSF data. Average $K_{d,\,app}$ values from triplicate measurements are shown for the respective protein and ligand. The marginal asymmetric confidence interval at a 95% confidence level (CI_{95}) for each $K_{d,\,app}$ value was estimated as suggested by Paketurytė *et al.*[2]

Protein species	$K_{d,\,app}$ (mM)	CI_{95}
scCSGal-1-6H M120L wt	3.14	[1.09, 8.16]
scCSGal-1-6H M120L E137AzK	5.05	[1.79, 13.16]
scCSGal-1-6H M120L E138AzK	8.50	[5.70, 12.72]
scCSGal-1-6H M120L R141AzK	5.13	[2.13, 11.93]
scCSGal-1-6H M120L Q142AzK	3.83	[1.66, 8.54]
scCSGal-1-6H M120L N144AzK	0.89	[0.40, 1.87]
scCSGal-1-6H M120L N50AzK	10.00	[3.84, 97.23]
scCSGal-1-6H M120L N192AzK	1.25	[0.17, 6.61]

Supporting References

(1) Rusconi, F.; Belghazi, M. Desktop prediction/analysis of mass spectrometric data in proteomic projects by using massXpert. *Bioinformatics* **2002**, *18* (4), 644-645.

(2) Paketurytė, V.; Petrauskas, V.; Zubrienė, A.; Abian, O.; Bastos, M.; Chen, W. Y.; Moreno, M. J.; Krainer, G.; Linkuvienė, V.; Sedivy, A.; Velazquez-Campoy, A.; Williams, M. A.; Matulis, D. Uncertainty in protein-ligand binding constants: asymmetric confidence intervals versus standard errors. *Eur. Biophys. J.* **2021**, *50* (3-4), 661-670.
